# B cell activation and plasma cell differentiation are inhibited by de novo DNA methylation

**DOI:** 10.1038/s41467-018-04234-4

**Published:** 2018-05-15

**Authors:** Benjamin G. Barwick, Christopher D. Scharer, Ryan J. Martinez, Madeline J. Price, Alexander N. Wein, Robert R. Haines, Alexander P. R. Bally, Jacob E. Kohlmeier, Jeremy M. Boss

**Affiliations:** 1 Department of Microbiology & Immunology, Emory University School of Medicine, 1510 Clifton Rd., Rm 3001, Atlanta, GA 30322 USA; 2 Present Address: Department of Hematology and Medical Oncology, Emory University School of Medicine, 1701 Uppergate Drive, WCI 4060 C, Atlanta, GA 30322 USA; 3 Present Address: Department of Medicine, Emory University School of Medicine, 1648 Pierce Dr. NE, Atlanta, GA 30307 USA; 4 Present Address: Department of Microbiology and Immunology, Yerkes National Primate Research Center, Emory University School of Medicine, 954 Gatewood Rd NE, Suite 3052, Atlanta, GA 30329 USA

## Abstract

B cells provide humoral immunity by differentiating into antibody-secreting plasma cells, a process that requires cellular division and is linked to DNA hypomethylation. Conversely, little is known about how de novo deposition of DNA methylation affects B cell fate and function. Here we show that genetic deletion of the de novo DNA methyltransferases *Dnmt3a* and *Dnmt3b* (Dnmt3-deficient) in mouse B cells results in normal B cell development and maturation, but increased cell activation and expansion of the germinal center B cell and plasma cell populations upon immunization. Gene expression is mostly unaltered in naive and germinal center B cells, but dysregulated in Dnmt3-deficient plasma cells. Differences in gene expression are proximal to Dnmt3-dependent DNA methylation and chromatin changes, both of which coincide with E2A and PU.1-IRF composite-binding motifs. Thus, de novo DNA methylation limits B cell activation, represses the plasma cell chromatin state, and regulates plasma cell differentiation.

## Abstract (web summary)

DNA methylation is known to contribute to B cell differentiation, but de novo methylation has not been studied in this context. Here the authors use a conditional Dnmt3a/b knockout mouse to map the function of de novo DNA methylation in B cell differentiation and the development of humoral immunity.

## Introduction

Appropriate regulation of B cell function is essential for humoral immunity and helps prevent antibody-dependent autoimmune diseases and B cell malignancies. Humoral immunity is maintained by mutually antagonistic transcription factor programs that either maintain B cell identity or promote plasma cell differentiation^1^. Upon stimulation, naive B cells rapidly proliferate while simultaneously amplifying and modulating their gene expression program, resulting in distinct cell fates and functions^2–6^. How gene expression programs are both remodeled and propagated across the many rounds of cellular division during B cell differentiation is not well understood. Epigenetic mechanisms, such as DNA methylation, have the potential to control gene expression and cell identity through mitosis^7^. Such is the case in B cells, where DNA hypomethylation is coupled to activation, proliferation, differentiation, and gene regulation^6,8–11^. Data thus far suggest that B cells undergo extensive and targeted DNA hypomethylation upon activation, but it is not known if de novo DNA methylation is also important for B cell fate and function.

DNA methylation is catalyzed by DNA methyltransferases, which in mammals occur primarily on the 5′-position of cytosine in the context of CpG dinucleotides^12^. DNA methylation represses transcription in promoters and mutagenic repetitive elements. Transcriptional enhancers are demarcated with intermediate amounts of DNA methylation^13,14^, where demethylation is enforced by transcription factor occupancy^14,15^. Highly expressed genes harbor high levels of gene-body DNA methylation^16^, which helps prevent spurious transcription^17,18^. DNA methylation is maintained through mitosis by the maintenance methyltransferase Dnmt1, which reciprocally methylates hemi-methylated CpGs formed during DNA replication^19^. This process is essential for mammalian development^19^, hematopoiesis^20,21^, lymphocyte maturation^22,23^, and differentiation^8,22,24^. Deposition of de novo DNA methylation by Dnmt3a and Dnmt3b is also required for mammalian development^25^ and when deleted in hematopoietic stem cells restricts B cell development^26,27^, but how it contributes to the molecular programming, differentiation, and function of mature B cells is not well understood.

To test the hypothesis that de novo DNA methylation is important for mature B cell function, *Dnmt3a* and *Dnmt3b* were conditionally deleted from B cells (Dnmt3-deficient) in mice. Dnmt3-deficient mice have phenotypically normal B cell development and maturation in the bone marrow, spleen, and lymph nodes, and mature follicular B cells show few molecular defects. Upon antigenic stimulation, Dnmt3-deficient mice have enlarged germinal center and plasma cell responses by a cell autonomous mechanism coupled to gene dysregulation, a failure to gain de novo DNA methylation, and repress the chromatin state in bone marrow plasma cells. Thus, Dnmt3-dependent DNA methylation restricts B cell activation and plasma cell differentiation.

## Results

### B cell development is independent of Dnmt3a and Dnmt3b

To conditionally delete both de novo DNA methyltransferases in B cells, mice containing the PC and ENV conserved catalytic domains of *Dnmt3a*^28^ and *Dnmt3b*^29^ flanked by *loxP* sites (fl) were crossed to mice that expressed the B-cell-specific *Cd19 Cre*-recombinase (*Cd19*^*cre*^)^30^. *Cd19*^*cre*^ is expressed at the pro-B cell stage, resulting in *Cre*-mediated recombination in pre-B cells^30^. Resultant mice showed genomic deletion of the catalytic sites of *Dnmt3a* and *Dnmt3b* in B cell lineages; whereas *Cre*-deficient littermate controls (*Dnmt3a*^fl/fl^*Dnmt3b*^fl/fl^) did not (Supplementary Fig. 1a). Dnmt3-deficient (*Cd19*^cre/+^*Dnmt3a*^fl/fl^*Dnmt3b*^fl/fl^) and -sufficient (*Dnmt3a*^fl/fl^*Dnmt3b*^fl/fl^) mice contained similar frequencies of bone marrow B220^+^CD43^−^ B cells (Supplementary Fig. 1b), as well as similar frequencies of bone marrow B220^mid^CD43^−^IgM^−^ pre-B cells, B220^mid^CD43^−^IgM^+^ immature B cells and B220^hi^CD43^−^IgM^+/lo^ mature B cells^31^ (Supplementary Fig. 1c). Likewise, Dnmt3-deficient and -sufficient mice had a similar frequency of splenic B220^+^CD43^−^ B cells, as well as B220^+^CD23^+^CD21^int/−^ follicular B cells and B220^+^CD23^−^CD21^hi^ marginal zone B cells^32^ (Supplementary Fig. 1d, e). Thus, no major cellular defects in B cell development and maturation were observed when *Dnmt3a* and *Dnmt3b* are deleted in CD19^+^ B cells.

### Dnmt3-dependent control of humoral immune responses

To test the role of de novo DNA methylation during B cell differentiation, B cells were differentiated ex vivo using both a T-cell-independent stimuli composed of lipopolysaccharide, interleukin 2, and interleukin 5 (LPS + IL-2 + IL-5), and a stimulus that mimics T-cell-dependent activation composed of CD40 ligand, interleukin 4, and interleukin 5 (CD40L + IL-4 + IL-5). Dnmt3 deficiency resulted in a higher frequency of GL7^+^-activated B cells as compared to Dnmt3-sufficent controls (Supplementary Fig. 2a). This result was not attributable to increased cellular division measured by cell trace violet dilution or a difference in cell death determined by cell viability dye exclusion, as both were equivalent between Dnmt3-sufficient and -deficient B cells (Supplementary Fig. 2b, c).

**Fig. 1:**
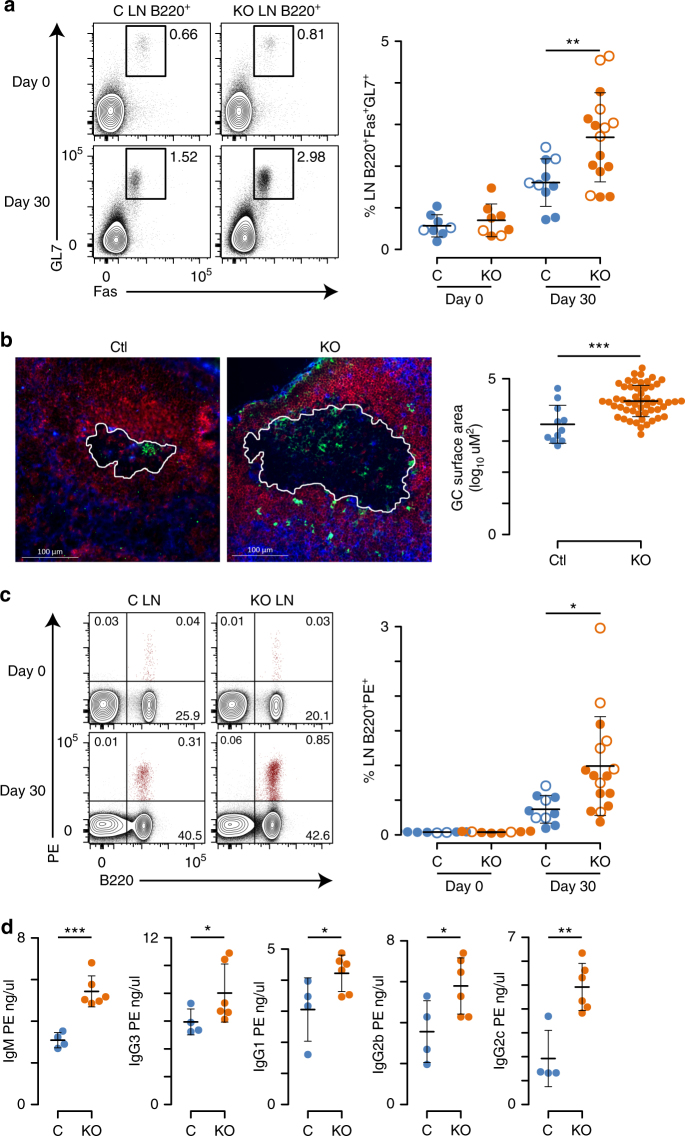
Increased germinal center B cell expansion in Dnmt3-deficient mice. **a** Germinal centers identified using Fas and GL7 expression on inguinal and periaortic lymph node (LN) B220^+^ B cells in naive mice (day 0) and PE-CFA immunized mice (day 30) in *Cd19*^*cre/+*^*Dnmt3a*^*fl/fl*^*Dnmt3b*^*fl/fl*^ mice (KO) and littermate controls (C). **b** Immunofluorescence of lymph nodes 30 days after PE-CFA immunization showing T cells (blue; Thy1.2), IgD B cells (red; IgD), and activated B cells (green; GL7). The germinal center area is outlined in white and a 100 μM scale is shown (bottom left). Quantitation of germinal center area is shown (right). **c** B220 expression and PE-binding of LN lymphocytes from naive mice (day 0) and PE-CFA immunized mice (day 30). Flow cytometry data are lymphocyte size gated and CD11b^−^. **d** Serum PE-specific antibodies for C and KO mice 30 days post-immunization. Mean and standard deviation shown on beeswarm plots and *P* values are calculated using a two-sided *t*-test (*P < 0.05, **P < 0.01, ***P < 0.001). Data are from either two, day 0 experiments with eight mice each and two, day 30 experiments with 9 mice and 17 mice (**a**, **c**) or from one, day 30 experiment with 10 mice (**b**, **d**), where open circles represent females and closed circles males and no sex differences were observed in multivariate analysis

The contribution of Dnmt3 enzymes to B cell activation was measured in vivo with subcutaneously immunization with phycoerythrin emulsified in complete Freud’s adjuvant (PE-CFA). PE-CFA immunization allows for the tracking of PE-specific B cell responses in the draining inguinal and periaortic lymph nodes, where the primary immune reaction occurs^33–35^. Analysis of lymph nodes provides a view of follicular B cells that is not confounded by marginal zone and B1b B cell subsets found in the spleen. Analysis of these lymph nodes in unimmunized mice revealed that ~0.8% of B220^+^ B cells were GL7^+^Fas^+^ germinal center B cells in both Dnmt3-sufficient and -deficient mice (Fig. 1a). At 30 days after PE-CFA immunization, the number of germinal center B cells increased twofold in control animals and fourfold in Dnmt3-deficient mice (Fig. 1a). Immunofluorescence performed on lymph nodes from PE-CFA-immunized mice indicated that this was at least partially due to an increase in germinal center size in Dnmt3-deficient mice as compared to littermate controls (Fig. 1b). PE binding of lymph node B220^+^ B cells revealed that unimmunized Dnmt3-sufficient and -deficient mice had very low and equivalent levels of B220^+^PE-specific B cells consistent with previous reports^34,35^. However, 30 days after PE-CFA immunization, Dnmt3-sufficient mice exhibited a ninefold increase (0.37%) in PE-binding B cells, whereas Dnmt3-deficient mice had a 25-fold increase (0.99%) (Fig. 1c). Staining for the proliferation marker, Ki-67 indicated equivalent levels of expression on total B220^+^ B cells, GL7^+^Fas^+^ germinal center B cells, and B220^+^PE-specific B cells from Dnmt3-sufficient and -deficient mice (Supplementary Fig. 2e, f), suggesting that these cells had similar levels of proliferation. Furthermore, serum antibody titers showed increased levels of PE-specific immunoglobulin 30 days after PE-CFA immunization in Dnmt3-deficient mice compared to controls (Fig. 1d).

**Fig. 2:**
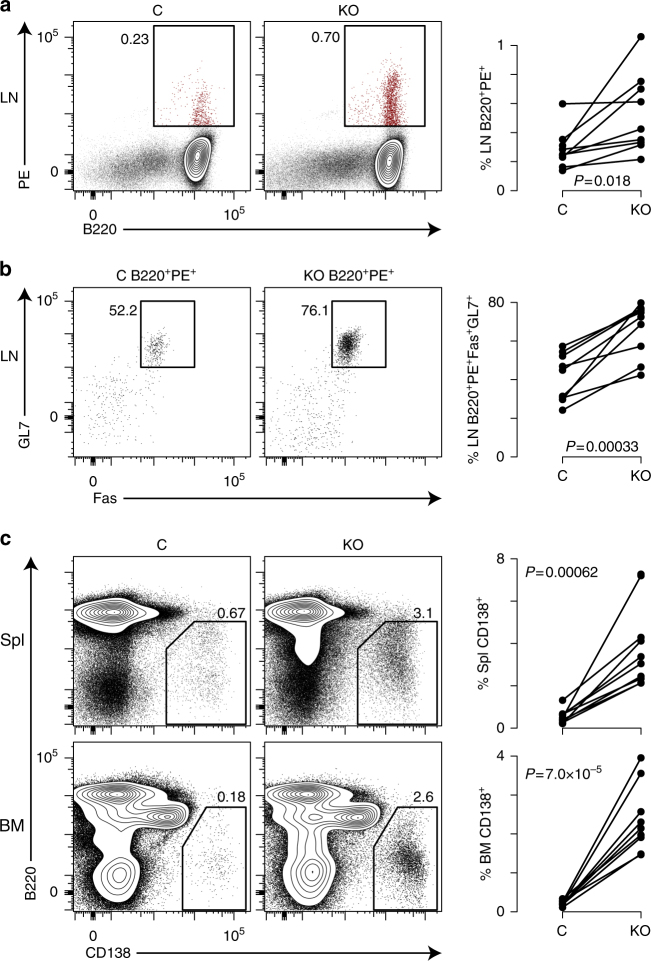
Cell autonomous control of B cell proliferation and differentiation. **a** B220 expression and PE binding of control (C) (CD45.1^+^CD45.2^+^ *Dnmt3a*^fl/+^*Dnmt3b*^fl/+^) and KO (CD45.2^+^ *Cd19*^cre/+^*Dnmt3a*^fl/fl^*Dnmt3b*^fl/fl^) cells in the inguinal and periaortic lymph nodes (LN) 30 days after PE-CFA challenge. **b** Fas and GL7 expression on B220^+^PE^+^ LN cells in **a**. **c** CD138 and B220 expression on C and KO cells in the spleen (Spl; top) and bone marrow (BM; bottom) of chimeric mice. All data are lymphocyte size gated and CD11b^−^CD11c^−^F4/80^−^Thy1.2^−^. *P* values are calculated using a paired *t*-test. Data represent two experiments with four and five mice per experiment with four male and five female mice

Cell autonomous effects of *Dnmt3a* and *Dnmt3b* on germinal center B cell responses were measured using bone marrow chimeras made by transferring a 1:1 ratio of CD45.2^+^ Dnmt3-deficient (*Cd19*^*cre/wt*^*Dnmt3a*^*fl/fl*^*Dnmt3b*^*fl/fl*^) and CD45.2^+^CD45.1^+^ Dnmt3-sufficient (*Dnmt3a*^*fl/+*^*Dnmt3b*^*fl/+*^) bone marrow cells into lethally irradiated CD45.1^+^ C57B/6J hosts. Six weeks after bone marrow reconstitution, mice were challenged with PE-CFA and analyzed 30 days later. Chimeric mice had approximately equal numbers of CD45.2^+^ Dnmt3-deficient and CD45.1^+^CD45.2^+^ Dnmt3-sufficient cells in the lymph nodes (Supplementary Fig. 3a). Bone marrow chimeras displayed greater frequencies of PE-specific B220^+^ B cells in Dnmt3-deficient vs. -sufficient cells relative to total chimeric cells (Fig. 2a). Additionally, of the PE-specific B220^+^ B cells, more of the Dnmt3-deficient cells were GL7^+^Fas^+^ germinal center in phenotype than their Dnmt3-sufficient counterparts (Fig. 2b). Analysis of CD138^+^ plasma cells in the spleen and bone marrow revealed a sharp increase in Dnmt3-deficient CD138^+^ plasma cells as compared to Dnmt3-sufficient cells (Fig. 2c). Together, these data show that *Dnmt3a* and *Dnmt3b* function to limit the humoral immune response, and the number of activated B cells and differentiated plasma cells through a cell autonomous mechanism.

### Dnmt3-dependent repression of B cell activation genes

**Fig. 3:**
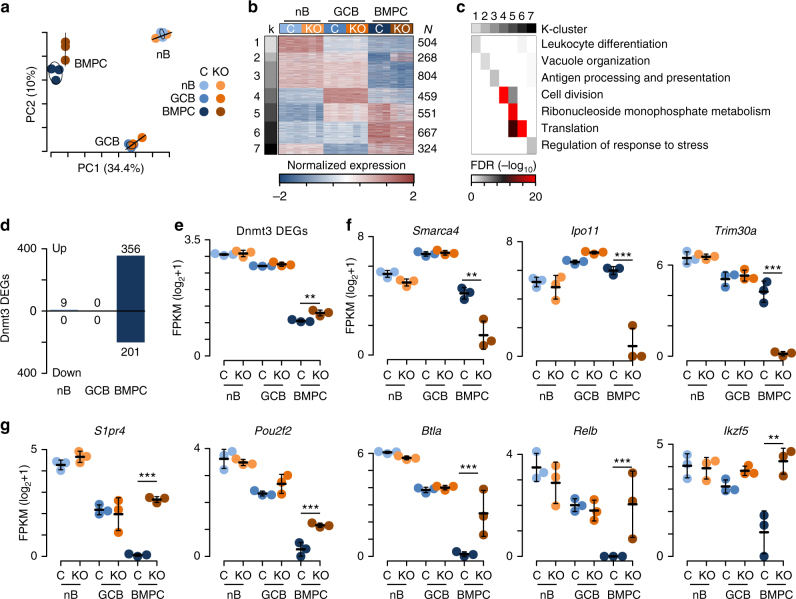
Dysregulation of plasma cell gene expression without Dnmt3a and Dnmt3b. **a** Principle component analysis of gene expression. The percentage in parenthesis is the proportion of variation explained by each component and 95% confidence intervals for each group are drawn. **b** K-means clustering of differentially expressed genes organized into seven categories uniquely. Samples are represented by columns and genes by rows. **c** Gene ontology analysis of genes contained in the K-clusters from **b**. Select ontologies are shown, and the significance of each ontology in each K-cluster (represented by columns) is denoted by color. **d** Number of Dnmt3-specific differentially expressed genes (DEGs) found between Dnmt3-sufficient and -deficient naive B cells (nB), germinal center B cells (GCB), and bone marrow plasma cells (BMPC). Genes upregulated in Dnmt3-deficient cells are shown above and those downregulated are shown below. **e** Average level of expression of all Dnmt3-specific differentially expressed genes shown in **d**. (**f**, **g**) Examples of Dnmt3 differentially expressed genes both downregulated (**f**) and upregulated (**g**) in Dnmt3-deficient BMPC. **FDR ≤ 0.01, ***FDR ≤ 0.001; mean and standard defviation are shown **f**, **g**. Data are from 3 experiments and 24 mice where nB were isolated from 6 mice, GCB were from 12 mice (2 mice were pooled per sample), and BMPC were from 6 mice. Each cell type contained 4 female and 2 male (nB, GCB) or 4 male and 2 female (BMPC) mice split evenly between the genotypes. Differentially expressed genes had an FDR ≤ 0.01 and a fold change ≥ 2

To determine how Dnmt3a and Dnmt3b influence B cell activation and differentiation, the gene expression program was determined for B220^+^GL7^−^Fas^−^ naive follicular B cells (nB) from the inguinal and periaortic lymph nodes of unimmunized mice, as well as B220^+^PE^+^GL7^+^Fas^+^ PE-specific germinal center B cells (GCB) from the same lymph nodes, and CD138^+^ bone marrow plasma cells (BMPC) 30 days after PE-CFA immunization for both Dnmt3-deficient and -sufficient mice. Cells were isolated to high purity by FACS (Supplementary Fig. 3b, c) and RNA was extracted for gene expression analysis by RNA-seq. Principle component analysis of gene expression data clustered samples into cell-type-specific groups, where Dnmt3-sufficient and -deficient samples overlapped each other in nB and GCB, but were distinct for BMPC (Fig. 3a), suggesting that the BMPC gene expression program was partially Dnmt3-dependent. Comparison of gene expression changes between nB and GCB revealed similar changes in expression in both Dnmt3-sufficient and -deficient mice (Supplementary Fig. 4a, left), suggesting that gene regulation during GCB cell differentiation was only subtly altered in the absence of *Dnmt3a* and *Dnmt3b*. However, comparison of gene expression changes between nB and BMPC showed a dysregulation of downregulated genes in BMPC between Dnmt3-sufficient and -deficient animals as compared to nB (Supplementary Fig. 4a, right). Indeed, comparison of differentially expressed genes between cell types indicated that Dnmt3-sufficient BMPC repressed a larger set of genes than Dnmt3-deficient BMPC (Supplementary Fig. 4b and Supplementary Data 1). Expressions of *Dnmt1* and *Dnmt3l* were not significantly different between Dnmt3-sufficient and -deficient cells, indicating that the expressions of other DNA methyltransferases were not compensating for the lack of Dnmt3 function (Supplementary Fig. 4c).

To better understand Dnmt3-dependent gene regulation in the context of B cell differentiation, differentially expressed genes were organized by K-means clustering. Incrementing k from 2 through 6 revealed distinct classes of genes differentially regulated between cell types and when k was set to 7 it identified genes distinctly regulated between Dnmt3-sufficient and -deficient BMPC (Fig. 3b). K-means cluster 2 was the most dysregulated group between Dnmt3-sufficient and -deficient BMPC (4.9-fold change, Tukey’s adj. *P* = 1.32 × 10^−12^). It contained genes that were expressed in nB and GCB and downregulated in control BMPC but not in Dnmt3-deficient BMPC. Annotation of genes contained within each K-means cluster yielded processes consistent with cell-type function (Supplementary Data 2). Statistically significant gene ontology terms associated with B cell function were highlighted in Fig. 3c. For instance, nB expressed genes involved in leukocyte-mediated immunity (cluster 1; e.g., Ccr7 and *Eomes*), GCB expressed genes involved in cell division and somatic hypermutation (cluster 4; e.g., Mki67 and *Aicda*), and BMPC expressed genes involved in translation and ER stress at high levels (cluster 6; e.g., Xbp1). K-means cluster 2 genes, which failed to be repressed in Dnmt3-deficient BMPC to the same degree as Dnmt3-sufficient BMPC, were involved in vacuole and lysosome organization, nucleotide metabolism, and inflammatory processes (Supplementary Data 2), but also included many genes known to be involved in B cell receptor signaling such as *Btk*, *Nfatc*, *Relb*, and *Cd86*.

Differentially expressed genes between Dnmt3-sufficient and Dnmt3-deficient nB, GCB, and BMPC were determined. This revealed that very few genes were aberrantly expressed in Dnmt3-deficient nB and GCB. However, in Dnmt3-deficient BMPC, 356 genes were upregulated and 201 downregulated compared to those from Dnmt3-sufficient mice (Fig. 3d). Summarizing the gene expression level of all Dnmt3-specific differentially expressed genes showed that these genes were on average downregulated in GCB and BMPC relative to nB, but that in Dnmt3-deficient BMPC they failed to be repressed to the same level as in Dnmt3-sufficient BMPC (Fig. 3e). Gene ontology annotation of genes downregulated in Dnmt3-deficient versus -sufficient BMPC indicated reduced expression of genes that mediate chromatin remodeling (e.g., Smarca4 and Yeats2) and regulate nuclear transport of ribosomal proteins (*Ipo11* and *Tnpo1*) (Supplementary Data 2). Other downregulated genes were involved in inhibition of NF-κB signaling (*Trim30a*) and cell cycle checkpoint (*Chek2* and *Pidd1*) (Fig. 3f). Conversely, genes upregulated in Dnmt3-deficient BMPC as compared to control BMPC included genes involved lysosome organization, nucleic acid-templated transcription, and metabolic processes (Supplementary Data 2). Other notable genes include *S1pr4*, which encodes a homing receptor important for B cell localization; *Pou2f2*, which encodes Oct-2, a factor required for B cell proliferation^36,37^; and Btla, which encodes an inhibitory receptor that is upregulated upon lymphocyte activation^38^; Relb, which is part of an activating heterodimeric NF-κB transcriptional complex; and the lymphocyte-specific transcription factor *Ikzf5* (Fig. 3g). Thus, gene expression reflected cell-type function and was primarily Dnmt3-independent in nB and GCB, but Dnmt3-deficient plasma cells showed dysregulation of genes involved in chromatin and transcription, organelle organization, metabolism, and activation.

RNA-seq reads were annotated to the B cell receptor to investigate the immunoglobulin (Ig) heavy (IgH) and light (IgL) chain repertoires. Ig expression composed 4.0% and 2.7% of all mRNAs that mapped to known genes in nB and GCB, respectively. Not surprisingly, BMPC expressed very high levels of Ig genes, accounting for 47.4% of all mRNAs (Supplementary Fig. 5a). Analysis of Ig isotype showed that nB expressed more than 99% IgM and IgD isotypes as expected, whereas GCB and BMPC Ig transcripts were 79% and 70% class-switched, respectively (Supplementary Fig. 5b). PE-specific GCB primarily expressed IgG isotypes similar to that previously reported^35^, whereas BMPC also expressed IgA isotypes. IgH variable, diversity, and joining chains were also examined, which showed similar repertoires of the variable region between Dnmt3-sufficient and Dnmt3-deficient nB, PE-specific GCB, and BMPC (Supplementary Fig. 5c). Notably, PE-specific GCB were mostly derived from a VH1-81 rearrangement, which may reflect their initial binding specificity to PE.

### De novo DNA methylation in B cell differentiation

**Fig. 4:**
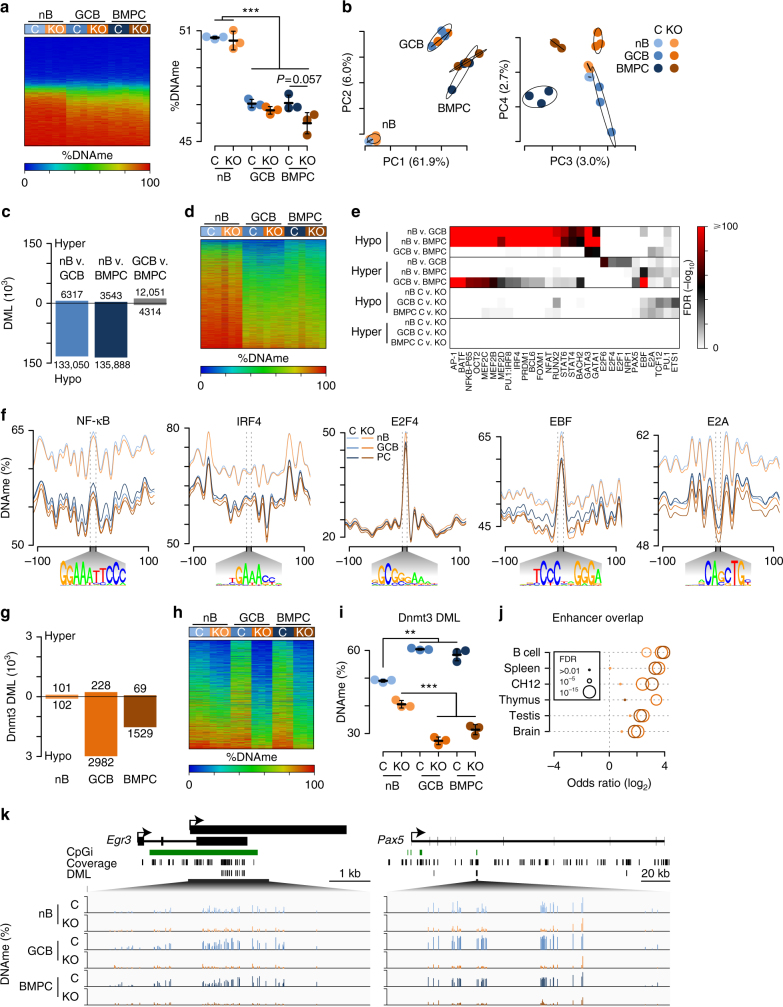
De novo DNA methylation during B cell differentiation. **a** Heat map of DNA methylation (DNAme) in lymph node B220^+^GL7^−^Fas^−^ naive B cells (nB), B220^+^PE^+^GL7^+^Fas^+^ germinal center B cells (GCB), and CD138^+^ bone marrow plasma cells (BMPC) in Dnmt3-sufficient (*Dnmt3a*^fl/fl^*Dnmt3b*^fl/fl^; C) and -deficient (*Cd19*^cre/+^*Dnmt3a*^fl/fl^*Dnmt3b*^fl/fl^; KO) mice (left) and average DNA methylation (right). **b** Principle components analysis of DNAme. The percentage in parenthesis is the proportion of variation explained by each component. **c** Number of differentially methylated loci (DML) between nB, GC, and BMPC cell types. **d** Heat map of all DML identified in **c**. **e** Analysis of motifs enriched within 50 bp of DML. The false discovery rate (FDR) significance is denoted by color (key right) and a full list can be found in Supplementary Data 6. **f** Plot of DNA methylation levels proximal to all genomic binding motifs for the indicated factors. **g** Number of DML between Dnmt3-deficient mice in nB, GCB, and BMPC relative to Dnmt3-sufficient cell types. **h** Heat map of loci identified in **g**. **i** Average DNA methylation for all loci determined in **f**. **j** Overlap of Dnmt3 DML with H3K4me1^+^H3K27ac^+^ enhancers in the designated cell types (light orange: nB; orange: GCB, brown: BMPC). **k** Examples of differentially methylated loci between Dnmt3-sufficient and Dnmt3-deficient cells. Scale is from 0 to 100% DNAme. **P ≤ 0.01, ***P ≤ 0.001, Student's two-sided *t-*test. Data were derived from 3 experiments and 24 mice, where nB were isolated from 6 mice, GCB were from 12 mice (2 mice were pooled per sample), and BMPC were from 6 mice. Each cell type contained four female and two male (nB, GCB) or four male and two female (BMPC) mice split evenly between the genotypes

To determine the functional targets of Dnmt3a and Dnmt3b in B cell differentiation, the same cells analyzed by RNA-seq were also subject to reduced representation bisulfite sequencing (RRBS)^39^, which resulted in 10× coverage at 1,658,881 CpGs per cell type. A heat map of DNA methylation levels revealed that GCB and BMPC had reduced levels as compared to nB (Fig. 4a), which is consistent with previous reports^6,9–11^. Comparison of the average DNA methylation levels showed that GCB and BMPC had considerably less methylation than nB (Fig. 4a, right). Principle component analysis of DNA methylation data showed components one and two separated samples by cell type, with principle component 1 representing the majority (61.9%) of variation and corresponding to the large number of hypomethylated loci in GCB and BMPC as compared to nB (Fig. 4b, left). Principle component four separated Dnmt3-sufficient and -deficient samples (Fig. 4b, right), indicating a reproducible component attributed to genetic ablation of *Dnmt3a* and *Dnmt3b*.

Differentially methylated loci (DML) between nB, GCB, and BMPC were determined using a statistical model that accounts for Dnmt3-specific effects^40^ (Supplementary Data 3). This identified over 130,000 hypomethylated CpG loci in GCB and BMPC as compared to nB, but only 6317 and 3543 loci that gained DNA methylation in GCB and BMPC, respectively (Fig. 4c). Comparison of GCB and BMPC indicated 12,051 loci that were more methylated in BMPC than GCB and 4314 loci that were less methylated (Fig. 4c). A heat map of all cell-type DNA methylation differences showed that the vast majority of these changes were hypomethylated loci in GCB and BMPC as compared to nB, and GCB contained a set of loci more demethylated than in BMPC (Fig. 4d). Venn diagrams of cell-type DML indicated that most hypomethylated loci in GCB and BMPC as compared to nB were shared, whereas hypermethylated loci in GCB were mostly distinct from those in BMPC (Supplementary Fig. 6a). Comparison of these DNA methylation changes with those observed in dividing B cells responding to the T-cell-independent antigen LPS^6^, indicated that 25% of demethylation events in GCB and BMPC were common to day 3 LPS-induced CD138^+^ plasmablasts, but only ~1% of loci hypermethylated in GCB and BMPC were shared with LPS-induced plasmablasts (Supplementary Fig. 7a). The few loci hypermethylated in response to LPS, gained DNA methylation in Dnmt3-sufficient GCB and BMPC, but not in Dnmt3-deficient cells (Supplementary Fig. 7b). This identifies a common DNA hypomethylation phenomenon in B cell activation, whereas de novo DNA methylation varies more between antigen-type, tissue, and/or time.

Cell-type-specific DML were compared to histone modifications in nB^41–43^, which indicated DML lacked histone 3 lysine 4 tri-methylation (H3K4me3), a modification found at promoters. DML hypermethylated in BMPC relative to nB and GCB had higher levels of histone 3 lysine 4 mono-methylation (H3K4me1) and histone 3 lysine 27 acetylation (H3K27ac), modifications that denote active enhancers^44^ (Supplementary Fig. 6b). Comparison of cell-type DML to active enhancers (demarcated by H3K4me1, H3K27ac, and no H3K4me3) in B cells, splenocytes, the lymphoma cell line CH12, and disparate tissues, including thymus, testis, and brain found that hypomethylated DML in BMPC and GCB as compared to nB preferentially overlapped active enhancers in B cells, splenocytes, and CH12 cells (Supplementary Fig. 6d). Hypermethylated DML in BMPC as compared to nB or GCB were also enriched for active enhancers in the same cell types, but those hypermethylated in GCB were not (Supplementary Fig. 6d). Loci hypermethylated in GCB as compared to nB contained higher levels of the repressive modification histone 3 lysine 27 tri-methylation (H3K27me3). H3K27me3 is deposited by the polycomb complex enzyme EZH2, which is necessary for germinal center B cell formation^45^, plasma cell differentiation^41^, and known to mechanistically recruit the DNA methyltransferases^46^ (Supplementary Fig. 6b). Together, these data indicate that hypomethylated regions during B cell differentiation are common in GCB and BMPC and co-occur at enhancers; whereas, hypermethylated regions are more distinct between GCB and BMPC, with the former coinciding with polycomb-marked regions and the later at enhancers.

Specific transcription factors potentially affected by these DNA methylation changes were investigated by identification of motifs proximal to DML. Regions demethylated in GCB and BMPC versus nB overlapped binding motifs of transcription factors important for B cell activation, such as AP-1 (e.g., BATF), NF-κB, OCT-2, MEF2-B, as well as factors required for differentiation, such as PU.1-IRF8, IRF4, BLIMP1 (encoded by *Prdm1*), BCL6, and BACH2 (Fig. 4e and Supplementary Data 4). The aforementioned activation factors more significantly overlapped regions demethylated in GCB relative to BMPC; whereas regions more demethylated in BMPC than GCB were enriched for GATA3 and GATA1 binding motifs. Regions hypermethylated in GCB relative to nB were enriched for E2F factors 1, 4, and 6, as well as NRF1; whereas BMPC hypermethylated loci were enriched for Pax5 and EBF1 motifs, and to a lesser extent the basic helix-loop-helix (bHLH) factors E2A and TCF12, and ETS factors PU.1 and ETS1 (Fig. 4e). DNA methylation levels relative to all genomic binding motifs showed that GCB and BMPC generally had less DNA methylation than nB, and that more subtle differences existed between GCB and BMPC (Fig. 4f). For instance, GCB had less DNA methylation than BMPC proximal to the binding site of activation factors NF-κB, MEF2-B, and OCT-2, whereas GCB and BMPC DNA methylation levels were about equivalent at differentiation factors IRF4, BLIMP1, and BCL6, but GATA1 had less DNA methylation in BMPC than GCB (Fig. 4f and Supplementary Fig. 6e). Interestingly, the binding sites of E2F factors 1, 4, 6, and NRF1 had equivalent levels of DNA methylation in all cell types and a local increase in DNA methylation at their motif sites, which contain a CpG (Fig. 4f and Supplementary Fig. 6e). These data indicate that transcription factors leave a considerable and organized imprint in the methylome of GCB and BMPC, which were delineated by subtle demethylation differences between their respective activation and differentiation transcription factors.

Comparison of DML between Dnmt3-sufficient and -deficient cell types indicated that relatively few differences exist in nB, but that GCB and BMPC had substantially more DNA methylation differences with the vast majority of CpGs having less DNA methylation in Dnmt3-deficient cells (Fig. 4g). Dnmt3-deficient cells failed to gain DNA methylation at a significant number of loci as illustrated in the heat map of Dnmt3-specific DML (Fig. 4h). Indeed, the average DNA methylation of these Dnmt3-specific loci showed that they gained DNA methylation in GCB and BMPC of Dnmt3-sufficient mice relative to control nB, but lost DNA methylation in the same cell types of Dnmt3-deficient mice (Fig. 4i). Comparison of these Dnmt3-specific loci with B cell histone modifications indicated that they occurred in regions with H3K4me1, H3K27ac, and H3K36me3 (Supplementary Fig. 6c). Consistent with those regions hypermethylated during B cell differentiation, Dnmt3-dependent DML preferentially overlapped active enhancers common to different cell and tissue types (Fig. 4j). Dnmt3-specific DML overlapped binding motifs of EBF1, bHLH factors E2A and TCF12, and ETS factors PU.1 and ETS1 (Fig. 4e). Dnmt3-sufficient cells had more DNA methylation at the genome-wide binding sites of these factors than their Dnmt3-deficient counterparts (Fig. 4f and Supplementary Fig. 6e). Examples of Dnmt3-dependent DML were common in CpG-rich regions, found at regulators of growth and B cell identity including *Egr3* and *Pax5*, respectively (Fig. 4k). In fact, the most differentially methylated locus in *Pax5* was previously identified as an enhancer element that controls *Pax5* expression during B lymphopoiesis^47^. Thus, while the majority of DNA methylation changes were losses associated with B cell activation and differentiation, there were several thousand loci that underwent de novo DNA methylation in germinal center B cells and bone marrow plasma cells.

### Dnmt3-dependent chromatin accessibility in plasma cells

**Fig. 5:**
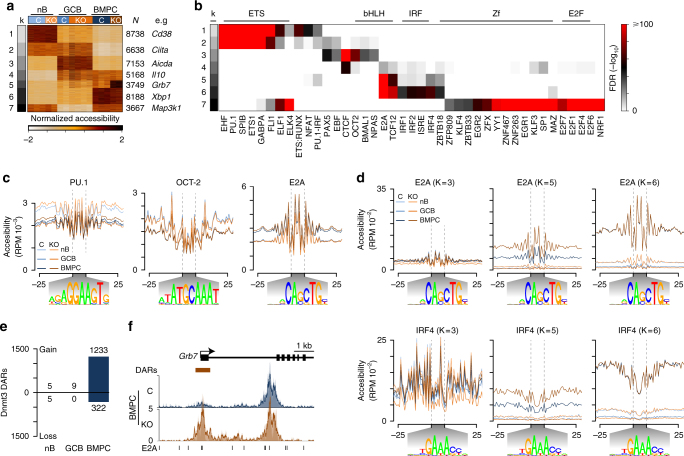
Increased chromatin accessibility in Dnmt3-deficient CD138^+^ BMPC. **a** K-means clustering of differential ATAC-seq peaks in B220^+^GL7^−^Fas^−^ naive B cells (nB), B220^+^PE^+^GL7^+^Fas^+^ PE-specific germinal center B cells (GCB), and CD138^+^ BMPC in *Cd19*^cre/+^*Dnmt3a*^fl/fl^*Dnmt3b*^fl/fl^ (KO) and littermate control (C) mice. The number of peaks in each cluster is denoted on the right. **b** Enrichment of HOMER transcription factor binding motifs in different K-means clusters shown in **a**. **c** Accessibility footprinting of ATAC-seq data shown for all PU.1, OCT-2, and E2A binding motifs genome-wide. Motifs are shown below. **d** Accessibility footprinting at E2A (top) and IRF4 (bottom) binding motifs that fall in specific K-means regions. **e** Number of differentially accessible regions (DARs) between Dnmt3-deficient (KO) and -sufficient (C) mice for nB, GCB, and BMPC. **f** Genome plot of regions containing differentially accessible regions (DARs) around *Grb7*. E2A transcription factor bind motifs are shown below. Data were derived from 3 experiments and 22 mice, where nB were isolated from 5 mice, GCB were from 12 mice (2 mice were pooled per sample), and BMPC were from 5 mice. Mice were either all male (nB), 4 female and 2 male split between the genotypes (GCB), or all female (BMPC)

To determine if de novo DNA methylation functionally influences chromatin structure in B cell differentiation, chromatin accessibility was determined for nB, GCB, and BMPC from both Dnmt3-sufficient and -deficient mice using the assay for transposase accessible chromatin sequencing (ATAC-seq)^48^. Hierarchical clustering and principle component analysis of accessible regions segregated samples by cell type indicating the largest variation in chromatin accessibility reflected cell-type-specific programming (data not shown). Differentially accessible regions between cell types and Dnmt3-sufficient and -deficient cells were determined and categorized by K-means clustering, revealing pervasive accessibility changes between nB, GCB, and BMPC (Fig. 5a and Supplementary Data 5). These changes were largely consistent between Dnmt3-sufficient and -deficient cell types, and regions of accessibility were found proximal to lineage factors that define nB, GCB, and BMPC, such as CD38, AID, and XBP-1, respectively (Fig. 5a). However, there was a set of regions that exhibited increased accessibility in Dnmt3-deficient BMPC (Fig. 5a, cluster 5).

Accessible regions in each K-means cluster were interrogated for overrepresentation of known transcription factor motifs, which identified increased accessibility of ETS binding sites in nB (e.g., PU.1 and SPI-B); proliferation factors, such as OCT-2 (encoded by *Pou2f2*) in GCB; and basic helix-loop-helix (bHLH, e.g., E2A) and interferon regulatory factor (IRF, e.g., IRF4) motifs enriched in BMPC (Fig. 5b and Supplementary Data 4). Indeed, genome-wide identification of putative PU.1, OCT-2, and E2A sequence binding motifs combined with ATAC footprinting showed that nB, GCB, and BMPC had greater chromatin accessibility at PU.1, OCT-2, and E2A binding motifs genome-wide, respectively (Fig. 5c). Accessibility footprints present in specific K-means clusters were also interrogated. In this analysis, increased accessibility was observed at several factors, including at E2A sites in BMPC (K = 6) as compared to GCB (K = 3) (Fig. 5d). Conversely, accessibility was remodeled at several sites, such as IRF4, where the motif footprint becomes focused depending on the K-cluster examined (e.g., GCB K = 3 v. BMPC K = 6; Fig. 5d). These results reflect the dramatic remodeling of the epigenome during B cell differentiation.

Accessibility differences between Dnmt3-deficient and Dnmt3-sufficient nB, GCB, and BMPC were determined, revealing few differences in nB and GCB, but that Dnmt3-deficient BMPC had 1233 more accessible and only 322 less accessible regions as compared to Dnmt3-sufficient BMPC (Fig. 5e), suggesting that Dnmt3-deficient BMPC had a general increase in accessibility. These regions largely overlapped those in K-means cluster 5 (see Fig. 5a) and were enriched for transcription factor binding motifs of the bHLH, ETS, IRF, and ETS-IRF composite families (Supplementary Data 4). This was visible in the genome-wide transcription factor footprints, where Dnmt3-deficient BMPC had a subtle increase in accessibility at E2A (bHLH) and PU.1 (ETS) binding motifs as compared to Dnmt3-sufficient BMPC (Fig. 5c). These differences in accessibility appear to be specific to a subset of regions. For example, accessibility footprints of E2A and IRF4 motifs showed substantial differences between Dnmt3-sufficient and -deficient BMPC at their respective motif binding sites in K-means group 5, but not at other K-means groups (Fig. 5d). One region that harbored such increases in accessibility and contained multiple E2A binding sites could be found at the promoter of the growth factor receptor gene *Grb7* (Fig. 5f). These data highlighted the exquisite regulation of chromatin accessibility between nB, GCB, and BMPC, and suggest that without de novo DNA methylation, BMPC accumulates an excess of accessible regions at binding motifs of both B cell (e.g., PU.1) and plasma cell (e.g., IRF4 and E2A) factors.

### Dnmt3-dependent repression of plasma cell gene expression

**Fig. 6:**
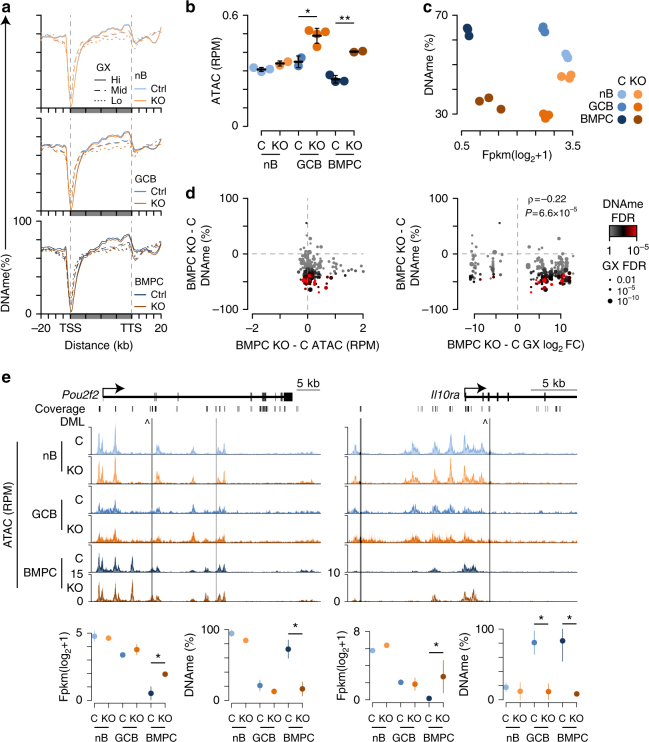
De novo DNA methylation accompanies gene repression in BMPC. **a** Average DNA methylation (DNAme) relative to the transcription start site (TSS) and transcription termination site (TTS) of genes stratified by expression level in lymph node B220^+^GL7^−^Fas^−^ naive B cells (nB; top), B220^+^PE^+^GL7^+^Fas^+^ germinal center B cells (GCB; middle), and CD138^+^ BMPC (bottom) for both *Cd19*^cre/+^*Dnmt3a*^fl/fl^*Dnmt3b*^fl/fl^ (KO; orange) and littermate control (C; blue). **b** Average ATAC-seq accessibility measured at Dnmt3-dependent differentially methylated CpGs. **c** Average gene expression and DNAme at Dnmt3-specific differentially expressed genes (*N* = 90) with proximal differentially methylated loci (*N* = 210). **d** Scatterplot of accessibility change by DNA methylation change for Dnmt3 differentially methylated loci (left), and differentially expressed genes and methylated loci from **c** in BMPC. Spearman's correlation coefficient (*ρ*) and significance of correlation are shown. **e** Examples of differentially expressed genes and methylated loci at the *Pou2f2* and *Il10ra* loci are shown. A schematic of the gene is shown (top) with examples of gene expression and DNA methylation changes shown below. The carrot (^) denotes the CpG shown below. Data are from 5 experiments and 36 mice as described (Figs. 3–5). Mean (**a**, **c**, **d**, **e**) and standard deviation are shown (**b**, **e**)

DNA methylation corresponds with gene expression, where actively transcribed genes are depleted of DNA methylation at promoters and enhancers, but contain high levels of gene-body DNA methylation^16^. Gene-body DNA methylation accumulates as elongating RNA polymerase II recruits SetD2 to catalyze H3K36me3, which is in turn bound by the PWWP domains of Dnmt3a and Dnmt3b^16,18^. Indeed, stratification of genes in nB, GCB, and BMPC showed that highly expressed genes in each cell type had a larger depletion of DNA methylation at promoters and more gene-body DNA methylation (Fig. 6a). This was similar in Dnmt3-sufficient and -deficient cell types, although Dnmt3-deficient cells had slightly less DNA methylation, which was most apparent at highly expressed genes.

To better understand how de novo DNA methylation impacts chromatin accessibility and gene regulation, Dnmt3-DML were annotated for chromatin accessibility and location relative to the closest gene (Supplementary Data 7). This revealed that Dnmt3-deficient GCB and BMPC gained chromatin accessibility at these CpG loci, whereas Dnmt3-sufficient controls did not (Fig. 6b), indicating that de novo DNA methylation represses the chromatin state at these loci. Comparison of CpG loci and genes found 213 DML in proximity to 91 differentially expressed genes, which was more than expected (*P* *=* 4.2 × 10^−6^). The average expression of these genes and DNA methylation at these loci showed that Dnmt3-deficient GCB and BMPC failed to gain DNA methylation at these loci and Dnmt3-deficient BMPC failed to repress gene expression to the same extent as their Dnmt3-sufficient counterparts (Fig. 6c). Inspection of individual gene/loci showed that a subset of Dnmt3-specific DML were associated with accessibility gains in BMPC and the DNA methylation of these same loci were negatively correlated with gene expression changes in Dnmt3-deficient BMPC (Fig. 6d). This suggests that DNA methylation at these regions serves to repress chromatin accessibility and inhibit transcription in BMPC. Indeed, several regions near key B cell activation genes, such as *Pou2f2* (encodes Oct-2), *Cd86*, *Nfatc1 Ebf1*, and *Il10ra*, failed to acquire DNA methylation at CpGs that coincided with increased accessibility at the boundaries of open chromatin, and aberrant expression in Dnmt3-deficient BMPC (Fig. 6e and data not shown). These data show that de novo DNA methylation preserves the chromatin architecture and is needed to fully repress the gene expression program of key B cell fate and activation genes during plasma cell differentiation.

## Discussion

Deletion of *Dnmt3a* and *Dnmt3b* during B cell development resulted in mature B cells that appeared normal with regard to cellular phenotype and frequency in the lymph nodes, spleen, and bone marrow. This was further supported by molecular analyses that showed very few transcriptional and epigenetic changes in Dnmt3-deficient naive follicular B cells as compared to Dnmt3-sufficient counterparts and is consistent with a recent whole-genome bisulfite analysis^49^. However, immunization of Dnmt3-deficient mice resulted in expanded antigen-specific germinal center B cells and serum antibodies as compared to Dnmt3-sufficient animals. Bone marrow Dnmt3-deficient and -sufficient chimera mice corroborated these findings and showed that they occurred by a cell autonomous mechanism, and indicated that the Dnmt3 deficiency results in an accumulation of plasma cells in the spleen and bone marrow. Molecular analyses of Dnmt3-sufficient and -deficient naive B cells, germinal center B cells, and bone marrow plasma cells indicated that the largest differences in transcriptional and epigenetic programming occurred in bone marrow plasma cells. Specifically, these results indicated that Dnmt3-deficient plasma cells failed to gain DNA methylation at over a thousand loci and that a significant portion of these genes failed to be fully repressed. Annotation of differentially expressed, methylated, and/or accessible genes indicated that these genes were involved in B cell lineage specification (e.g., Ebf1), cytokine signaling (e.g., *Il10ra*), anatomical localization (e.g., *S1pr4*), and B cell activation (e.g., *Cd86*, *Pou2f2*, *Nfatc1*, and *Nfatc2*). Thus, these results demonstrate that de novo DNA methylation limits the B cell activation program and regulates plasma cell differentiation.

The observation that B cell development is largely undisturbed in the absence of the de novo DNA methyltransferases was somewhat surprising given the importance of these enzymes in mammalian development^25^. This may be explained by the fact that common lymphoid precursors gain copious amounts of DNA methylation^10,50^, a developmental step that occurs just before CD19 expression, which was used to delete Dnmt3a and Dnmt3b. Indeed, others who have deleted *Dnmt3a* and *Dnmt3b* using *Mb1-cre*, which is expressed earlier than *Cd19-cre*^51^, found that deletion of *Dnmt3a* and *Dnmt3b* resulted in precocious Igκ rearrangement^52^. We did not, however, observe skewing in the variable chain usage of the IgH or Igκ in naive B cells, germinal center B cells, or bone marrow plasma cells. Nonetheless, immunization of Dnmt3-deficient mice resulted in increased B cell activation and differentiation, a phenomenon that was more pronounced over extended antigenic challenge (i.e., 30 days) but was harder to discern over shorter time periods. This suggests that de novo DNA methylation may have relatively modest effects on gene expression during B cell differentiation, but as DNA methylation changes accumulate and are propagated through cell division they result in a skewing toward the plasma cell fate. Indeed, when *Dnmt3a*^26^ or *Dnmt3a* and *Dnmt3b*^27^ were deleted in hematopoietic stem cells, increases in self-renewal and progressive defects in hematopoiesis were most apparent after several months and multiple serial transplantations in competition with wild-type control cells. Likewise, analysis of bone marrow chimeras here showed that Dnmt3-deficient B cells had increased activation and accumulation of splenic and bone marrow plasma cells as compared to controls.

Previous molecular analyses indicated pervasive DNA hypomethylation apparent in both germinal center B cells and plasma cells, suggesting that this occurs during the initial stages of B cell activation and proliferation and is subsequently propagated to progeny cell fates^6,8–11^, consistent with the data generated here. There were, however, more modest gains in DNA methylation in germinal center B cells and plasma cells as compared to naive B cells. This de novo DNA methylation was largely Dnmt3-dependent and clustered in contiguous regions around genes that are normally downregulated during B cell differentiation, such as *Egr3, Ebf1, Il10ra, Nfatc2, Pax5*, and *Pou2f2*. Although a significant portion of these Dnmt3-dependent DNA methylation changes were associated with gene expression changes, many were only partially repressed, and the majority were not. This suggests that during plasma cell differentiation, DNA methylation is not the initiating mechanism of gene silencing, but rather transcription factors and other epigenetic mechanisms are employed to restrict gene expression and that Dnmt3-dependent DNA methylation is a complementary means of fully repressing expression at a subset of genes. Irrespective of antigen (PE-CFA vs. LPS), there is a global hypomethylation of ~10% of CpGs, with a significant portion of these regions overlapping regulatory elements. It is likely that the paucity of significant gains in DNA methylation observed in response to LPS^6^ was simply due to the short duration of the response (3 days). Therefore, de novo DNA methylation likely functions to reinforce the silencing of B-cell-specific genes.

Integrative analyses of gene expression, DNA methylation, and chromatin accessibility revealed the exquisite transcriptional and epigenetic regulation, as well as the contribution of de novo DNA methylation to germinal center B cell and plasma cell differentiation. While, little to no molecular differences were observed between Dnmt3-sufficient and -deficient naive B cells, Dnmt3-deficient germinal center B cells failed to accumulate methylation, yet there was little perturbation in gene expression and chromatin accessibility. This could be the result of selection of germinal center B cells for a specific gene expression program and/or it may indicate that the external stimulus from cytokines and antigen overpowers any effects on gene expression from the Dnmt3-dependent DNA methylation during the germinal center reaction. Alternatively, there may be something in the bone microenvironment and/or cognate core regulatory program that makes plasma cell gene expression more susceptible to influence of Dnmt3-dependent DNA methylation. In sharp contrast to germinal center B cells, there was a significant overlap and inverse correlation of DNA methylation and gene expression changes in bone marrow plasma cells. These Dnmt3-dependent DNA methylation changes occurred at regions that function as enhancers in a diverse set of cell types and overlapped binding motifs for EBF1, bHLH factors E2A and TCF12, and ETS factors PU.1 and ETS1. Moreover, this de novo DNA methylation repressed the chromatin state of individual CpGs, and Dnmt3-deficient plasma cells consistent with the Dnmt3-dependent DNA methylation motif analysis contained increased chromatin accessibility at E2A, PU.1, IRF4, and ZBTB18 binding motifs, transcription factors previously identified as regulated by DNA methylation, and/or the de novo DNA methyltransferases^52–54^. Indeed, the co-occurrence of de novo DNA methylation with the boundaries or “shores” of chromatin accessibility suggest that DNA methylation demarcates a larger epigenetic architecture of plasma cells. These results show that de novo DNA methyltransferases mediate important epigenetic programming differences that are necessary for limiting B cell activation, establishing the plasma cell gene expression program, and repressing plasma cell differentiation.

## Methods

### Ex vivo differentiation

B cells were isolated using immunomagnetic separation (Miltenyi Biotec) to negative sort splenic CD43^−^ B cells. Cells were labeled with CTV (ThermoFisher scientific) at a concentration of 20 × 10^6^ cells/ml in PBS. B cells for LPS differentiation were plated at 0.5 × 10^6^ cells/ml and provided an initial stimulation of LPS (20 µg/ml; Sigma #L2630), IL-2 (20 ng/ml; eBioscience #14-8021), and IL-5 (5 ng/ml; eBioscience #14-8051). Half doses of LPS and cytokines were given on every 24 h. CD40L-stimulated cells were plated at 0.2 × 10^6^ cells/ml and given daily stimulation with CD40L (100 ng/ml; R&D Systems), IL-4 (10 ng/ml; R&D Systems), and IL-5 (5 ng/ml; R&D Systems).

### Mice and PE-CFA immunization

*Dnmt3a*^*fl/fl*^ (*Dnmt3a*^*tm3.2Enl*^)^28^ and *Dnmt3b*^*fl/fl*^ (*Dnmt3b*^*tm5.1Enl*^)^29^ mice were previously described and crossed to Cd19^cre/wt^ mice (B6.129P2(C)-*Cd19*^*tm1(cre)Cgn*^/J (Jackson stock # 006785). Mice were between 8 and 12 weeks of age at the start time of experiments and were housed by the Emory Division of Animal Resources. Approximately equal ratios of male and female Dnmt3-sufficient and Dnmt3-deficient mice were used for all experiments and are defined in the figure legends. All protocols were approved by the Emory Institutional Animal Care and Use Committee (IACUC) and included a power analysis for proposed experiments. Investigators were not blinded and no animals were excluded from analysis unless otherwise noted. Experiments were balanced such that similar numbers of mice were included in each group.

PE-CFA immunization was prepared similar to that previously described^34^. Briefly, R-Phycoerythrin (PE; Prozyme) was emulsified in a 1:1 ratio of Complete Freud’s Adjuvant (CFA; Sigma-Aldrich) and PBS such that 15 μg of PE was used per 100 μl CFA:PBS. Mice were immunized subcutaneously with 100 μl of PE-CFA in the flank.

### Flow cytometry and cell isolation

Splenic and lymph node cell suspensions were made by mechanically forcing tissues through a 40 µm filter. Bone marrow cells were isolated from femurs and tibias by fracturing the bones in a mortar and pestle. Splenic and bone marrow red blood cells were lysed with ACK lysis buffer (0.15 M NH_4_Cl, 10 mM KHCO_3_, 0.1 mM EDTA) for 30 s prior to quenching the reaction with 4 volumes RPMI 1640 media (Corning Cellgro) supplemented with 10% heat-inactivated fetal bovine serum (Sigma-Aldrich), 1% MEM non-essential amino acids, 100 μM Na pyruvate (Sigma), 10 mM HEPES (Sigma), 0.0035% β-mercaptoethanol (Sigma-Aldrich).

Cells were washed and resuspended at 10^7^ cells/ml in PBS with 1% BSA and 2 mM EDTA and blocked with anti-Fc (anti-CD16/CD32) (Tonbo Biosciences, 2.4G2) at a final concentration of 0.25 μg/10^6^ cells for 15 min on ice, prior to staining. Flow cytometry panels included the following stains to remove auto-fluorescent cells: anti-CD11b (Tonbo Biosciences M1/70), anti-CD11c (Tonbo Biosciences N418), anti-CD90.2 (Tonbo Biosciences 30-H12), and anti-Ly6-G (Tonbo Biosciences 1A8) conjugated to FITC or APC-Cy7 each at a concentration of 0.25 μg/10^6^ cells. The following antibody fluorophore and dyes were used to assess cellular phenotype: anti-B220-PerCP-Cy5.5 or -PE-Cy7 (Tonbo Biosciences, RA3-6B2) at 0.05 μg/10^6^ cells; anti-CD43-FITC (BD #553270) at 0.125 μg/10^6^ cells; anti-CD138-PE, -BV421, or -BV711 (BD, 281-2) at 0.025 μg/10^6^ cells; anti-GL7-eFluor660 (eBioscience GL7) at 0.025 μg/10^6^ cells; anti-CD45.1-APC-Cy7 (Tonbo Biosciences A20); Viability Violet Stain (Life Technologies L34955), Ki-67-PE-Cy7 at 0.025 μg/10^6^ cells (BD Biosciences B56). Cells were stained for 30 min and fixed using 1% paraformaldehyde prior to analysis. For intracellular stains (Ki-67), cells were permeabilized with PBS with 1% BSA, 2 mM EDTA, 0.1% sodium azide, and 0.1% saponin and stained for 30 min prior to fixing in 1% paraformaldehyde. Staining panels included fluorescence minus one controls to ensure that correct compensation was applied, as well as isotype controls to assess non-specific staining. Representative flow gating strategies are shown in Supplementary Fig. 8. Flow cytometric analysis was conducted on a Becton Dickinson (BD) LSRII, and FCS files were exported using FACSDiva (v6.2). Analysis of flow cytometric data was conducted in R/Bioconductor (v.3.2.2) using the “flowCore” (v.1.36.9) package or FlowJo software (v9.7.6). Validation data for all antibodies used are available on the manufacturer’s website.

### Bone marrow chimera mice

For bone marrow cells CD45.2^+^ Dnmt3-sufficient (*Dnmt3a*^*fl/fl*^ *Dnmt3b*^*fl/fl*^) were bred to CD45.1^+^ C57BL/6J mice (B6.SJL-Ptprca Pepcb/BoyJ; Stock No: 002014) and CD45.1^+^ CD45.2^+^ *Dnmt3a*^*fl/+*^ *Dnmt3b*^*fl/+*^ offspring were used as controls for Dnmt3-deficient mice (CD45.2^+^ *Cd19*^*cre*^ *Dnmt3a*^*fl/+*^ *Dnmt3b*^*fl/+*^). Bone marrow cells were isolated from femurs and tibias of 8–12-week-old mice and red blood cells were lysed in ACK lysis buffer (0.15 M NH_4_Cl, 10 mM KHCO_3_ 0.1 mM EDTA). Dnmt3-sufficient and -deficient bone marrow cells were mixed 1:1 and tail vein injected into mice that were irradiated with 950 rads. Mice were fed antibiotic water containing 2% sucrose, 0.5 mg/ml neomycin, 0.0125 mg/ml polymyxin B for 7 days after irradiation and were immunized with PE-CFA 8 weeks after irradiation.

### Enzyme-linked immunosorbent assay

For determination of PE-specific IgM, IgA, IgG1, IgG2a, IgG2b, IgG2c, and IgG3 antibodies, flat-bottom ELISA plates (Evergreen Scientific) were coated with PE (Prozyme) diluted in borate-buffered saline to a concentration of 5 μg/ml. Plates were incubated overnight at 4 °C, washed, blocked with 3% non-fat dry milk for 2 h at room temperature, washed, and diluted sera samples were added. Following a second overnight incubation at 4 °C, plates were washed and horseradish peroxidase (HRP)-conjugated goat anti-mouse IgM, IgG1, IgG2b, IgG2c, and IgG3 (Southern Biotechnology) were applied. After a final wash, TMB ELISA peroxidase substrate (Rockland Immunochemicals) was applied to develop a color reaction, and optical density at 650 nm (OD_650_) was collected by a Synergy HT Multi-Mode Microplate Reader (BioTek). The first three wash steps were performed with PBS containing 0.05% Tween 20 while the final wash was performed with PBS. Antibody concentrations were determined by comparing antibody levels for experimental samples with the standard curves of purified mouse IgM, IgA, IgG1, IgG2a, IgG2b, IgG2c, and IgG3 (Southern Biotech). Significant differences in antibody levels between samples were determined using two-sample *t*-tests with a *P* value of 0.05 being considered significant.

### Immunofluorescence

OCT-embedded lymph nodes were sectioned at 7 microns onto positively charged glass slides and stored at −80 °C until staining. The slides were fixed for 10 min in 75:25 acetone/ethanol at room temperature. Sections were serum blocked for 30 min at room temperature with PBS containing 10% v/v normal mouse serum, 10% v/v normal rat serum, 10% v/v normal donkey serum, 2% w/v bovine serum albumin, 0.05% w/v sodium azide, and 1 μg/ml anti-CD16/32 clone 2.4G2 (clone 2.4G2 ATCC HB-197). Biotin blocking was performed with an Endogenous Biotin Blocking Kit (ThermoFisher E21390) per the manufacturer’s instructions. Slides were then stained with a primary antibody cocktail of anti-CD90.2-FITC (1:100, clone 30-H12, Biolegend, 105306), GL7-Biotin (1:20, eBioscience, 13-5902-81), and anti-IgD-A647 (1:50, clone 11–26c.2a, Biolegend, 405708) in serum blocking buffer. Secondary stain consisted of anti-FITC-A488 (1:100, polyclonal, Life Technologies, A11090) and Strepavidin-Hilyte555 (1:20, Anaspec, 60666) in serum blocking buffer. Coverslips were mounted with Pro-Long Gold Antifade Mountant (ThermoFisher, P36930). Between each step, slides were washed with PBS 4× 30 s and dried on the grate of a BSC. Slides were cured overnight and imaged on a Zeiss Axio Observer A1 using Zen software (v2.0.0.0).

### mRNA-seq

mRNA-seq was performed from 5000 FACS-isolated cells for all samples. Cells were sorted using a FACS Aria (Becton-Dickson) into RPMI 1640 media (Corning Cellgro) supplemented with 10% heat-inactivated fetal bovine serum (Sigma-Aldrich), 1% MEM non-essential amino acids, 100 μM sodium pyruvate (Sigma), 10 mM HEPES (Sigma), 0.0035% β-mercaptoethanol (Sigma-Aldrich). Cells were then centrifuged at 500 × *g* for 10 min and media was decanted prior to resuspension in 600 μl of RLT buffer (Qiagen) with 1% β-mercaptoethanol and frozen at −80 °C. RNA was extracted with RNeasy microkit (Qiagen #74004) according to the manufacturer’s directions with on-column DNase digestion. RNA quality was checked on an Agilent Bioanalyzer and had an RNA Integrity Number greater than 7. Reverse transcription was done using the SMART-Seq v2 kit (Clontech) and adapters were added using the Nextera kit (Illumina). Libraries were quantitated and quality controlled using an Agilent Bioanalyzer and qPCR quantitation method (Kapa Biosystems), prior to pooling and 50 bp paired-end sequencing on a HiSeq 2500 (Illumina).

### mRNA-seq analysis

FASTQ files from mRNA-sequencing were quality trimmed using FastQC (v0.11.5; https://www.bioinformatics.babraham.ac.uk/projects/fastqc/) with the default settings. Quality trimmed FASTQ files were mapped to the mouse genome (mm9) using Tophat^55^ (v2.1.1) with the following settings: “-p 10 -N 2 --max-multihits 1 --read-gap-length 1”. Mapped BAM files were sorted with SAMtools (v0.1.19-96b5f2294a)^56^. Gene-level expression of UCSC KnownGenes^57^ were determined by calculating the number of reads that uniquely overlap gene exons using R/Bioconductor (v.3.2.3)^58^ and the “summarizeOverlaps” function of the GenomicAlignments package (v1.6.3)^59^. Differentially expressed genes (DEGs) were determined using EdgeR (v3.12.1)^60^, where a Benjamini–Hochberg FDR ≤0.05 and a twofold change in expression was required for significance. Only genes expressed at more than 1 FPKM in at least two samples were considered for differential analysis. Gene ontology of differentially expressed genes was performed using the R/Bioconductor GOStats package (v.2.36.0) and considered dependencies and similarities in calculating GO term *P* values, which were subsequently FDR corrected. Gene set enrichment analysis (GSEA)^61^ used the pre-ranked option based on the significance of differential expression determined by EdgeR. K-means clustering was performed using the “kmeans” function of the “stats” package in R/Bioconductor and used a maximum number of 50 iterations with 100 random sets determine K-groups.

Immunoglobulin heavy (IgH) chain and light chain expression as well as IgH isotype were determined by annotating reads to IMGT^62^ defined immunoglobulin genes. The percentage of Ig reads were calculated as the total number of reads mapping to Ig genes relative to all other reads mapped to UCSC KnownGenes.

### Reduced representation bisulfite sequencing

Reduced representation bisulfite sequencing (RRBS) was performed from 5000 cells isolated in parallel with those used for mRNA-seq. Cells were lysed in buffer (20 mM Tris (pH 8.0), 4 mM EDTA, 20 mM NaCl, and 1% SDS) with proteinase K and RNase A at 67 °C overnight. DNA was isolated using phenol–chloroform–isoamylalcohol and ethanol precipitation. DNA was split into separate *MspI* (New England Biolabs) and *TaqI* (New England Biolabs) digestions and incubated overnight with 1000 units of enzyme. Digested DNA was combined and cleaned up using a 1.8× solid phase reversible immobilization (SPRI) beads (Agencourt AMPure XP Beads; Beckman Coulter). Aliquot of 1 pg of lamda DNA (New England Biolabs, #N3011S) was spiked-in to each reaction as an unmethylated control. DNA was end-repaired and A-tailed using the Hyper Prep Kit (KAPA Biosystems) following the manufacturer’s protocol. Short Truseq compatible sequencing adapters that contained fully methylated cytosine residues synthesized (Integrated DNA Technologies) and ligated also using the Hyper Prep Kit (KAPA Biosystems). Adapter-ligated DNA was bisulfite treated using the EpiTect Bisulfite Kit (Qiagen), modifying the manufacturer’s protocol by increasing the denaturation temperature to 99 °C and extending the time from 5 to 10 min. Adapter-ligated bisulfite-treated libraries were amplified 12–13 times using HiFi Uracil + Polymerase (KAPA Biosystems) and library concentration was estimated using the qPCR library quantification kit (KAPA Biosystems) and size estimation by a high sensitivity DNA Bioanalyzer (Agilent Technologies). Libraries were sequenced using 50 bp paired-end reads on a HiSeq 2500 by the Genome Technology Center at New York University (NYU).

### RRBS analysis

RRBS FASTQ files were quality trimmed using FastQC (v0.11.5) and mapped to the mouse genome (mm9) using Bismark (v0.16.3)^63^ using the following options: “--bam --chunkmbs 1024 –multicore 8”. Mapped BAM files were sorted with SAMtools (v0.1.19-96b5f2294a)^56^ and methylation calls were extracted using the “Rsamtools” (v.1.22.0) and “data.table” (v.1.9.6) packages, as well as custom R scripts as previously described^6^. Coverage was determined for all CpGs with 10× coverage in each sample (*N* = 615,148) and in each group (*N* = 1,658,881). Differential analysis was performed on all CpGs w/ 10× coverage per group using dispersion shrinkage for sequencing (v2.10.0)^64^, where significance was determined by an FDR ≤0.05 and an absolute change in DNA methylation ≥20%. Heat maps, principle component analyses, and average methylation were performed on CpGs with coverage in each sample. The odds ratio of overlap of differentially methylated CpGs with enhancers in B cells, splenocytes, the lymphoma cell line CH12, thymus, testis, and brain tissue was determined using Fisher’s exact test as previously described^6^. Briefly, active enhancer regions were defined as regions that harbor histone modifications H3K4me1 and H3K27ac but lack H3K4me3^44^ as determined from published chromatin immunoprecipitation sequencing (ChIP-seq) experiments (Gene Expression Omnibus GSE51336^43^ and GSE51011^42^). Enriched regions were either those that were published by the ENCODE project^43^ (http://genome.ucsc.edu/cgi-bin/hgFileSearch?db=mm9) or were determined by MACS software (v1.4)^65^.

### The assay for transposase accessible chromatin sequencing

ATAC-seq was performed on 3000–5000 sorted cells similarly to that previously described^48,66^. Briefly, cells were pelleted at 500 × *g* for 10 min at 4 °C and resuspended in 50 μl nuclei lysis buffer (10 mM Tris pH 7.4, 10 mM NaCl, 3 mM MgCl_2_, 0.1% IGEPAL) and centrifuged at 500 × *g* for 30 min at 4 °C. Nuclei were resuspended in 22.5 μl of tagmentation DNA buffer (Illumina) with 2.5 μl tagmentation enzyme (Illumina) at 37 °C for 60 min. DNA was isolated by proteinase K digestion (2 μg) at 40 °C for 30 min followed by negative (0.6×) and positive (1.2×) size selection with SPRI beads (Agencourt AMPure XP, Beckman Coulter). ATAC-seq libraries were amplified 12–13 times with Hifi Polymerase (Kapa Biosystems) and quantitated by qPCR (Kapa Biosystems) and high sensitivity bioanalyzer (Agilent) and sequenced using 50 bp paired-end sequencing on an Illumina HiSeq 2500 (Illumina) by NYU Genome Technology Center.

### ATAC-seq analysis

ATAC-seq FASTQ files were quality trimmed using FastQC (v0.11.5) and mapped to the mouse genome (mm9) using Bowtie (v2.2.6)^67^, using the default options. Mapped SAM files were sorted and converted to BAM files with SAMtools (v0.1.19-96b5f2294a)^56^. Accessible regions were determined using MACS2 (v2.1.0.20151222)^65^ with the following options: “callpeak -g mm –q 0.01”. The union of all sample peaks was determined in R (v3.2.3)^58^ using the package “GenomicRanges” (v1.22.4)^59^. The number of reads in accessible regions was used to normalize the signal with the following equation:

1 $${\rm{RPM}} = {\rm{reads}} \times \frac{{10^6}}{{{\rm{total}}\;{\rm{reads}}\;{\rm{in}}\;{\rm{peaks}}}}$$

RPM denotes reads per million. As a quality-control metric the number of reads in peaks was determined and samples that did not contain 5–25% of reads in peaks were eliminated from subsequent analysis. This included one sample. Differentially accessible regions were determined using edgeR (v3.12.1)^60^ for all accessible regions, where an FDR ≤0.01 and a signal fold change ≥2 was required for significance. K-means clustering of differentially accessible regions was performed in R using the function “kmeans” with the parameters: “iter.max = 1000, nstart = 100”. K-means clusters were annotated for enrichment of transcription factor motifs by determining the genome-wide binding motifs of transcription factors defined by the software HOMER^68^, using the function “scanMotifsGenomeWide.pl” and then testing for overrepresentation in each K-means group using Fisher’s exact test. Transcription factor footprinting was calculated by counting only the base at which the ATAC-seq read began and centering it relative the transcription factor binding motifs determined above using custom R scripts available upon request.

### Integrative analysis

Meta-analysis of the DNA methylation levels across genes (stratified by expression) was calculated by annotating each CpG to the closest transcript and polynomial smoothing the average DNA methylation using the R function “loess” with the parameter: “span = 0.1”. The accessibility of cytosines in a CpG context was determined by calculating the normalized number of ATAC-seq reads that started on the cytosines of CpGs that are differentially methylated between Dnmt3-sufficient and Dnmt3-deficient cell types. Significance of overlap of differentially methylated CpGs and differentially expressed genes were determined by annotating each CpG to the closest transcript and determining the expected overlap given the number of differentially methylated CpGs in the context of total CpGs covered and number of differentially expressed genes. The significance of the inverse correlation between gene expression and DNA methylation was measured using Spearman’s *ρ* and significance was determined using ANOVA.

### Code availability

All codes are available upon request.

### Data availability

Sequence data that support the findings of this study have been deposited in the Gene Expression Omnibus with the primary accession code GSE89471.

## Electronic supplementary material

Supplementary Information

Description of Additional Supplementary Files

Supplementary Data 1

Supplementary Data 2

Supplementary Data 3

Supplementary Data 4

Supplementary Data 5

Supplementary Data 6
