# Supplementary material for: B cell activation and plasma cell differentiation are inhibited by de novo DNA methylation
Source: Nat Commun. 2018 May 15;9:1900. doi: 10.1038/s41467-018-04234-4 (PMC5953949; doi:10.1038/s41467-018-04234-4)
Supplement: Supplementary file 1 — Supplementary Information [file 41467_2018_4234_MOESM1_ESM.pdf]

Supplementary Information for

B cell activation and plasma cell differentiation are inhibited by de novo DNA methylation

Barwick *et al.*

Supplementary Figure 1

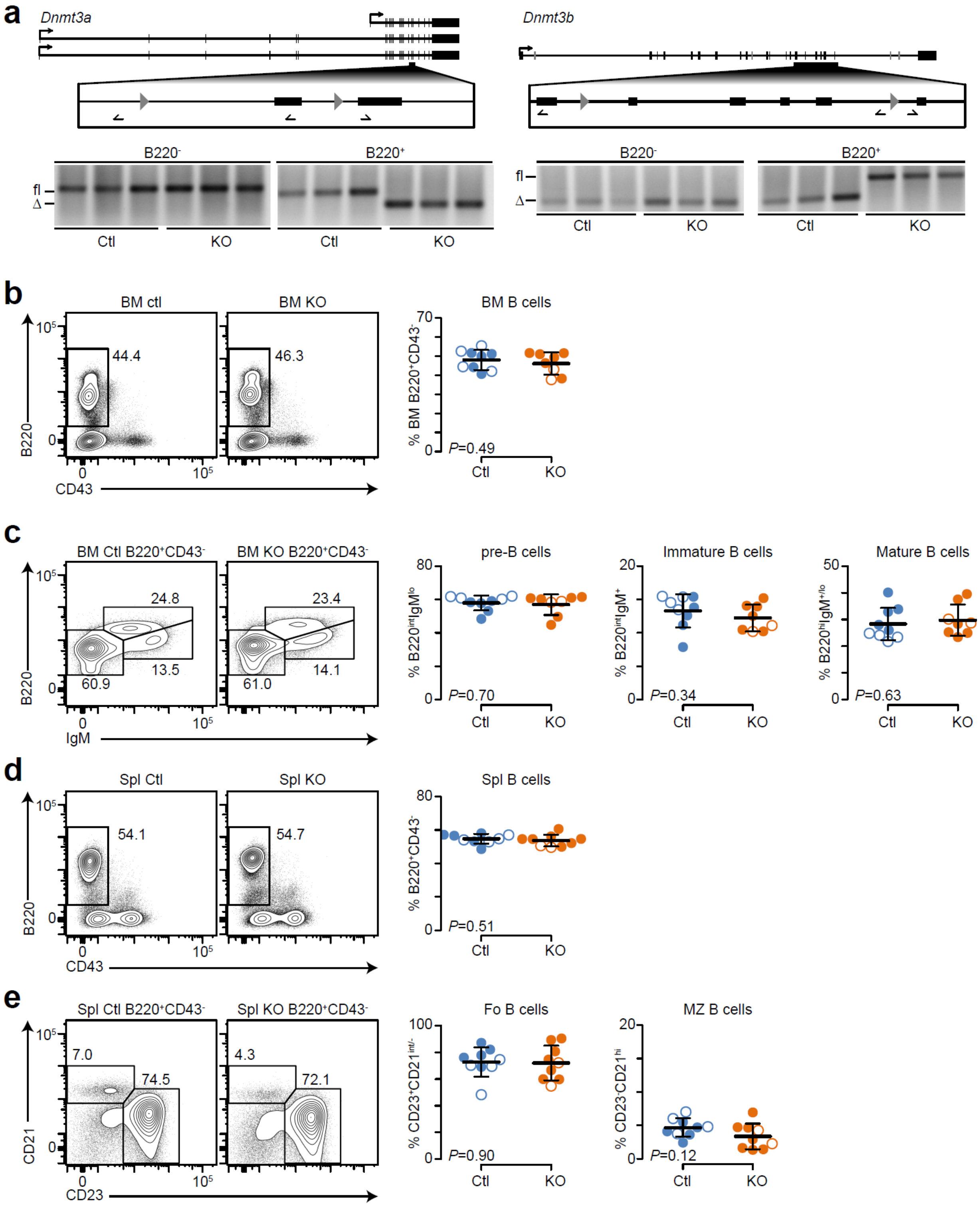

Supplementary Figure 1. B cell development is phenotypically normal in Dnmt3-deficient mice. **(a)** Schematic of *Dnmt3a* (left) and *Dnmt3b* (right) with regions flanked by *loxP* sites (gray triangles) enlarged. PCR primers (black arrows) were used to amplify the region and measure genomic

rearrangement in bone marrow B cells ($B220^+$) and non B cells ($B220^-$). Both Dnmt3-sufficient (*Dnmt3a*$^{fl/fl}$ *Dnmt3b*$^{fl/fl}$; Ctl) and Dnmt3-deficient (*Cd19*^{cre} *Dnmt3a*$^{fl/fl}$ *Dnmt3b*$^{fl/fl}$; KO) mice are shown. **(b)** B220 and CD43 expression in the bone marrow (BM) from representative Ctl and KO mice. Data are summarized showing the frequency of $B220^+CD43^-$ B cells in the BM (right). **(c)** IgM and B220 expression on $B220^+CD43^-$ gated bone marrow B cells shown in part **b**. The three gates represent $B220^{int}IgM^-$ pre-B cells, $B220^{int}IgM^+$ immature B cells and $B220^{hi}IgM^{+/lo}$ mature B cells. **(d)** B220 and CD43 expression in the spleen (Spl) from Ctl and KO mice. **(e)** CD23 and CD21 expression on splenic $B220^+CD43^-$ B cells. Quantitation of $CD23^+CD21^{int/-}$ follicular and $CD23^-CD21^{hi}$ marginal zone B cells are shown on the right. All data are lymphocyte size gated and $CD11b^-$. *P*-values were calculated using a two-sided *t*-test. Data are from two experiments with 6 and 7 mice per experiment (**a**) or 8 and 10 mice per experiment (**b-e**), female mice are denoted by open circles and male by closed circles. Mean and standard deviation are shown on summary beeswarm plots.

Supplementary Figure 2

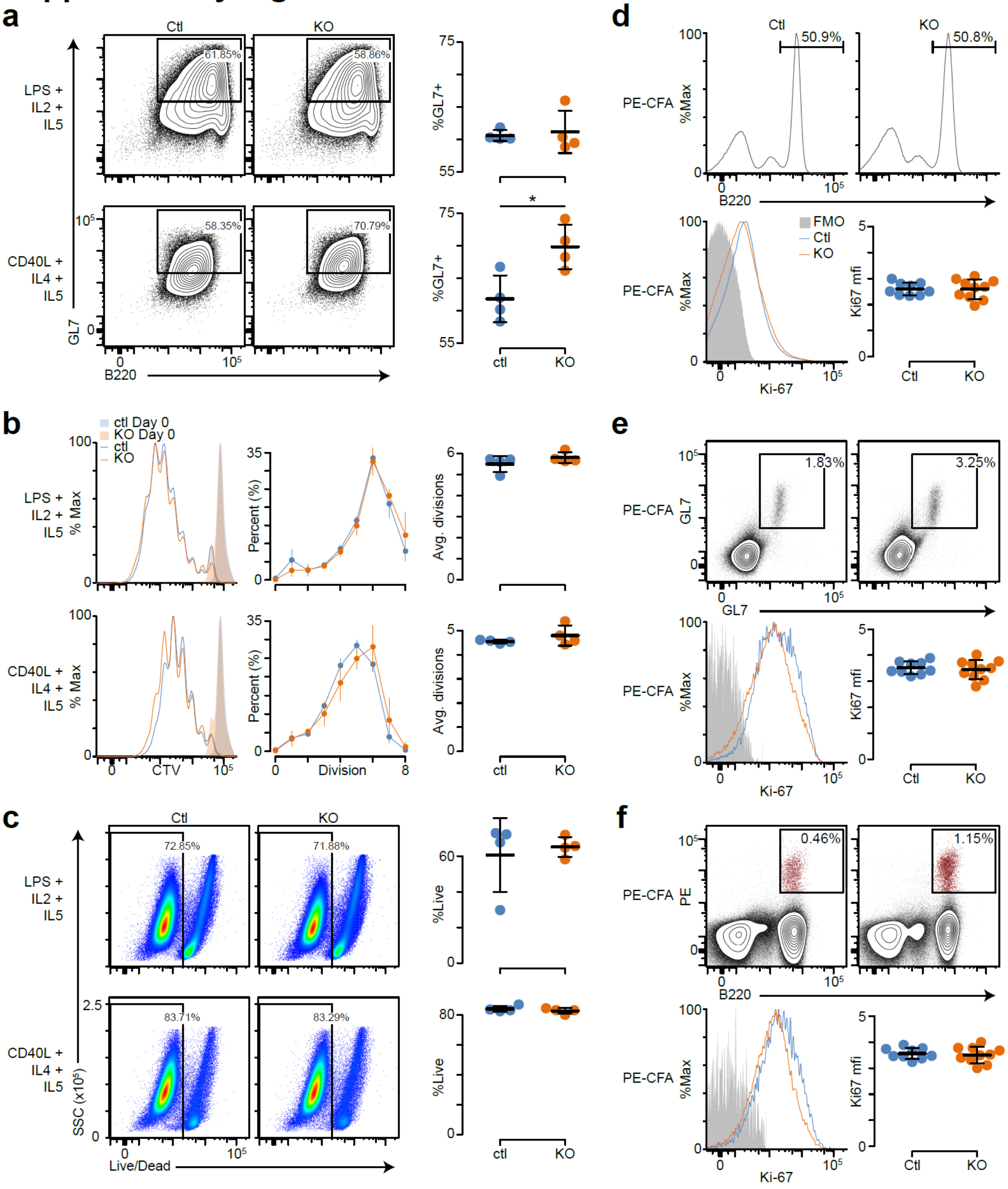

Supplementary Figure 2. Dnmt3-deficient B cells undergo increased activation *ex vivo* in response to T-cell stimuli. **(a)** B cell activation measured using GL7 reactivity for Dnmt3-sufficient (Ctl) and –deficient (KO) B cells stimulated *ex vivo* for 3 days using T cell-independent stimulus consisting of lipopolysaccharide, IL2, and IL5 (LPS+IL2+IL5; top), or CD40 ligand, IL4, and IL5 (CD40L+IL4+IL5; bottom). Quantitation is shown (right). **(b)** Cell division measured using cell trace violet (CTV) dilution.

Percent of cells by division is quantitated (middle) and the average number of divisions for each differentiation is shown (right). **(c)** Cell death was measured using a cell exclusion live/dead dye for both LPS+IL2+IL5 (top) and CD40L+IL4+IL5 (bottom) conditions and quantitated (right). **(d-f)** Ki-67 staining on total B220+ B cells (**e**), B220+GL7+Fas+ germinal center B cells (**e**), and PE-specific B cells (**f**), all 30 days after PE-CFA immunization. Representative B cells subsets for Ctl (top left) and KO (top right) mice, and Ki-67 staining (bottom left) are shown with mean fluorescence intensity (MFI) is quantitated (bottom right). Data are from one experiment with 8 mice including B cells from 4 Ctl and 4 KO mice (**a-c**) or one experiment with 19 mice including 9 Ctl and 10 KO (**d-f**). Mean and standard error are shown and *P*-values were calculated using a two-sided *t*-test (*P <0.05, **P < 0.01).

Supplementary Figure 3

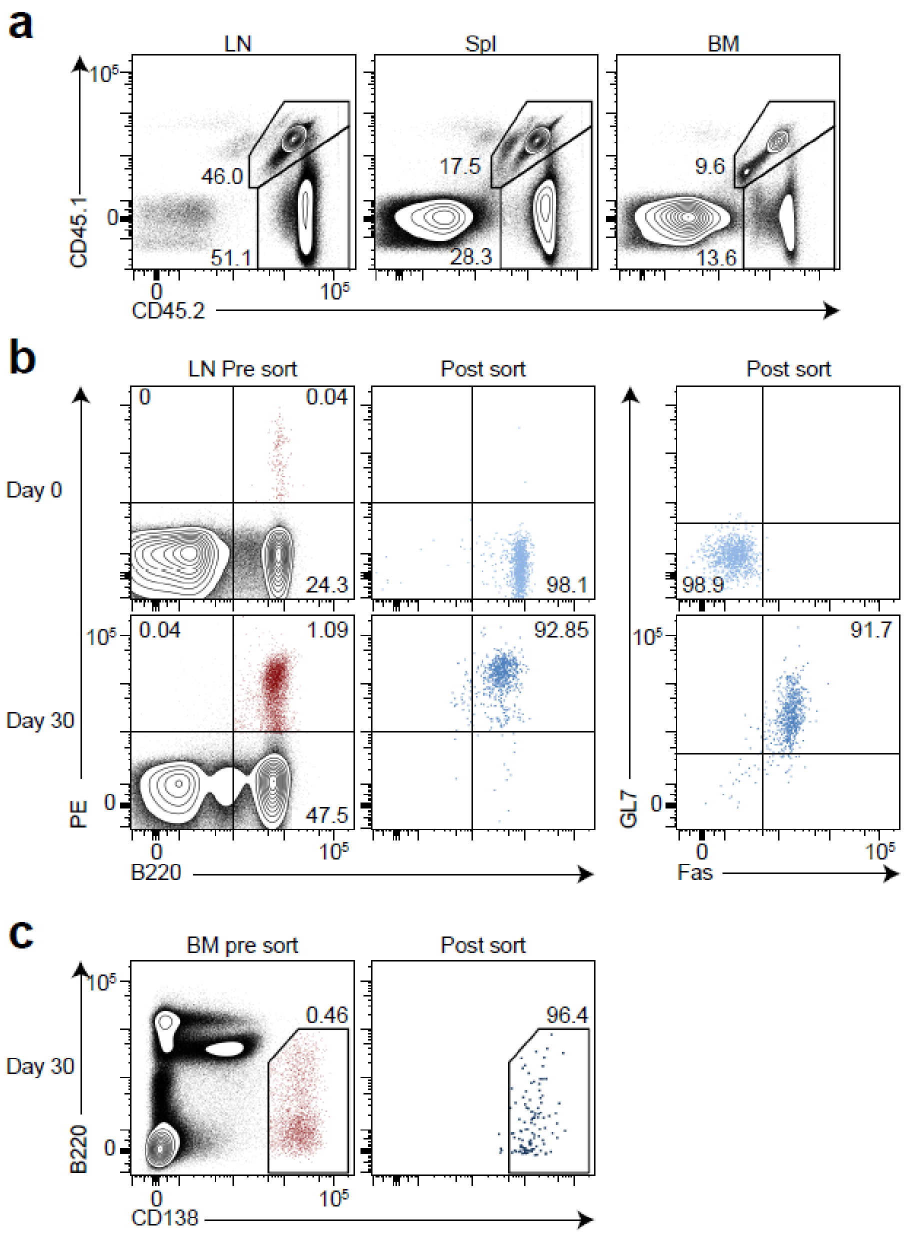

Supplementary Figure 3. Frequency of Dnmt3-sufficient and –-deficient cells in mixed bone marrow chimera mice and isolation of naive B cells (nB), germinal center B cells (GCB), and bone marrow plasma cells (BMPC) for molecular analyses. **(a)** CD45.1 and CD45.2 expression in the inguinal and periaortic lymph nodes (LN), spleen (Spl), and bone marrow (BM) of chimeric mice made from CD45.2^{+} *Cd19*$^{cre/+}$*Dnmt3a*$^{fl/fl}$*Dnmt3b*$^{fl/fl}$ (KO) and CD45.1^{+}CD45.1^{+} *Dnmt3a*$^{fl/+}$*Dnmt3b*$^{fl/+}$ (Ctl) bone marrow cells. **(b)** B220 expression and PE-binding in lymph nodes (left) and post sort populations (middle) as well as Fas and GL7 expression on B220^{+}PE^{+} B cells (right). **(c)** B220 and CD138 expression in bone marrow cells before (left) and after (right) sorting. Data are representative from two experiments with 5 and 4 mice per experiment (**a**) or 5 experiments where nB were isolated from 8 mice or 6 mice, GCB were

isolated from 14 mice (2 mice were pooled for RNA-seq and RRBS analyses), and BMPC were isolated from 8 mice or 6 mice.

Supplementary Figure 4

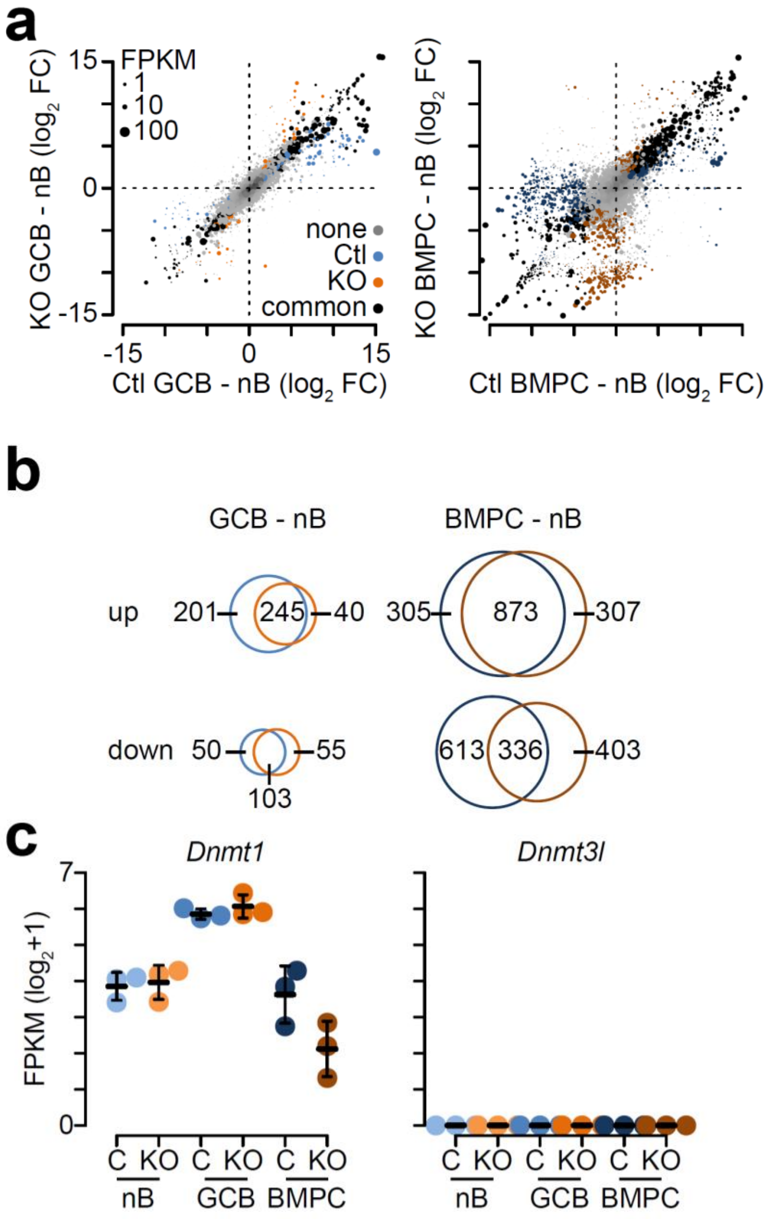

Supplementary Figure 4. Aberrant gene regulation in Dnmt3-deficient bone marrow plasma cells (BMPC). **(a)** Scatter plot of gene expression changes between naive B cells (nB) and germinal center B cells (GCB; left) and BMPC (right) for Dnmt3-sufficient (Ctl; x-axis) and -deficient (KO; y-axis) mice. The size of the dot represents the level of gene expression represented in fragments per kilobase per million reads (FPKM; see key top right) and differentially expressed genes between nB, GCB, and BMPC are denoted in blue (Ctl only), orange (KO only), or black (both Ctl and KO). **(b)** Venn diagram of differentially expressed genes between nB and GCB (left) or nB and BMPC (right). Both upregulated (top) and downregulated (bottom) genes are shown relative to nB. **(c)** Expression of DNA methyltransferases *Dnmt1* and *Dnmt3l* genes. Mean and standard deviation are shown. RNA-seq data

are from 18 samples from 24 mice and 3 experiments, where 3 KO and 3 Ctl samples were analyzed for nB, GCB, and BMPC and two mice were pooled for GCB. Each cell type contained 4 female and 2 male (nB, GCB) or 4 male and 2 female (BMPC) mice split evenly between the genotypes. Differentially expressed genes had an FDR ≤0.01 and a fold-change ≥2.

Supplementary Figure 5

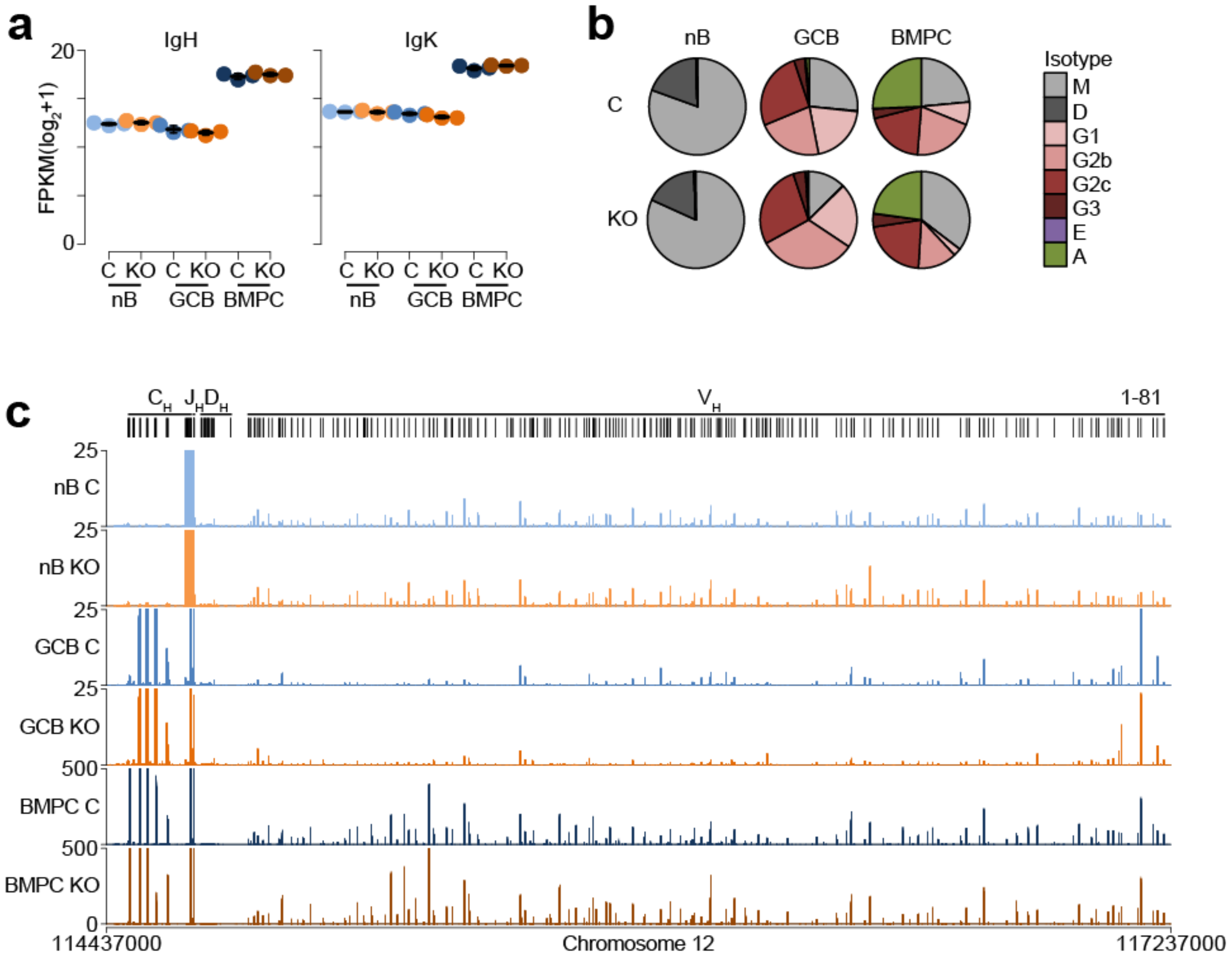

Supplementary Figure 5. Immunoglobulin repertoire, isotype, and expression. **(a)** Expression of Immunoglobulin heavy (IgH) and kappa light (IgK) chains for $B220^{+}GL7^{-}Fas^{-}$ naive B cells (nB) , $B220^{+}PE^{+}GL7^{+}Fas^{+}$ germinal center B cells (GCB) and $CD138^{+}$ bone marrow plasma cells (BMPC) in *Cd19*$^{cre/+}$*Dnmt3a*$^{fl/fl}$*Dnmt3b*$^{fl/fl}$ (KO) mice and littermate controls (C). **(b)** Isotype expression for IgH constant (C_H) chain in nB, PE-specific GCB, and BMPC in both KO and C mice. **(c)** Plot of IgH region showing average reads from nB, PE-specific GCB, and BMPC in both KO and C mice. PE-specific GCB are primarily of the V_H1-81 repertoire labeled above. Data in **b** and **c** represent the average of 3 biological replicates. RNA-seq data are from 18 samples from 24 mice and 3 experiments, where 3 KO and 3 Ctl samples were analyzed for nB, GCB, and BMPC and two mice were pooled for GCB. Each cell type contained 4 female and 2 male (nB, GCB) or 4 male and 2 female (BMPC) mice split evenly between the genotypes. Differentially expressed genes had an FDR ≤0.01 and a fold-change ≥2.

Supplementary Figure 6

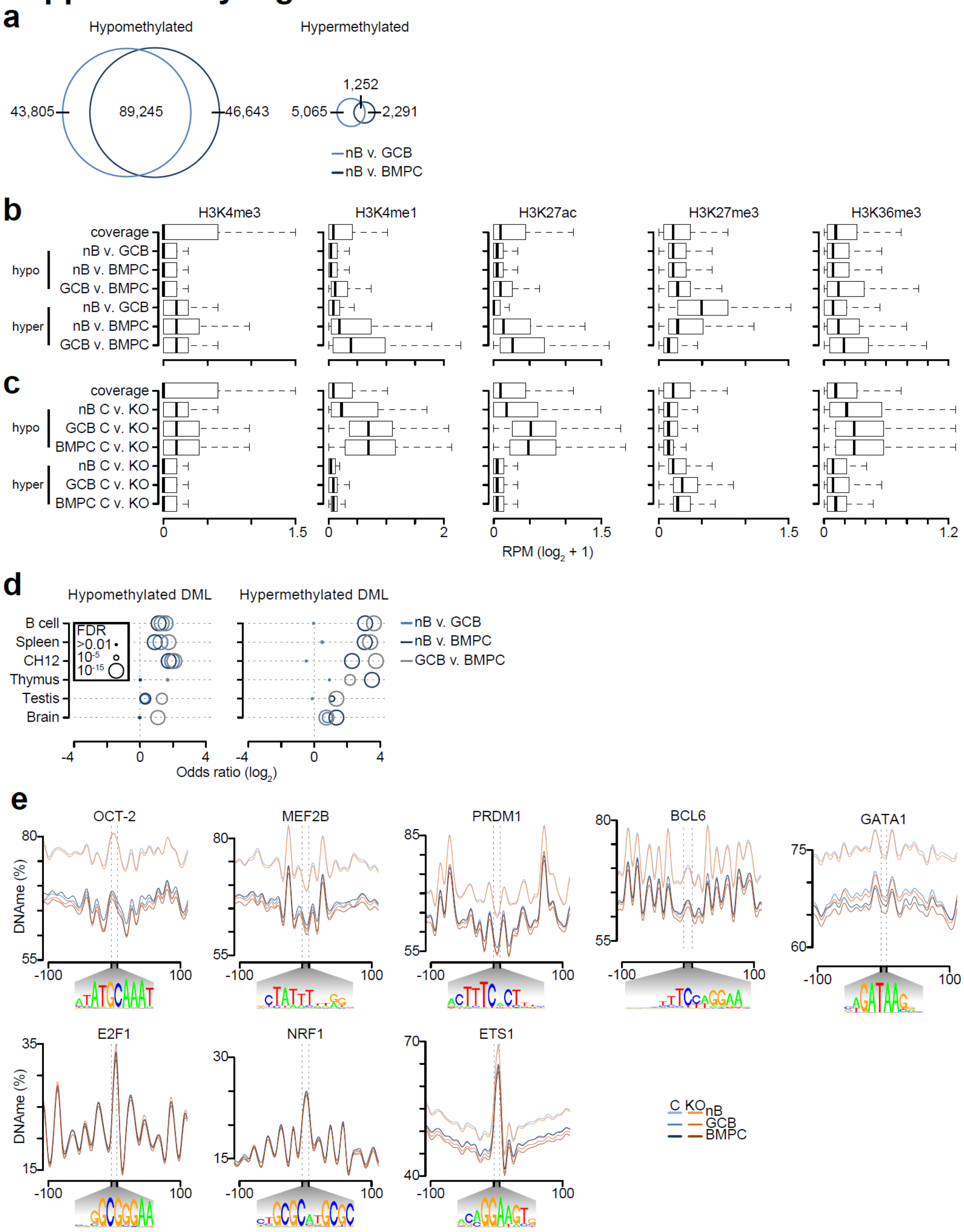

Supplementary Figure 6. DNA methylation changes correspond with enhancer elements and are organized around transcription factor binding motifs. **(a)** Venn diagram of DML hypomethylated (left) and hyper (right) in GCB (blue) and BMPC (dark blue) compared to nB. **(b-c)** Histone modification enrichment of H3K4me3, H3K4me1, H3K27ac (from Sabo *et al.* [42]), H3K27me3 (from Guo *et al.* [41]), and H3K36me3 (from ENCODE [43]) in B cells at cell-type (**b**) and Dnmt3-specific (**c**) DML. Hypomethylated and hypermethylated indicate the first comparison relative to the second. RRBS assay coverage is denoted (cov) and histone enrichment is represented in $\log_2$ reads per million (RPM) for a region covering the DML +/- 50 bp. **(d)** Odds-ratio of cell-type DML overlap with active enhancers (defined as regions H3K4me1+ H3K27ac+ and H3K4me3-) in B cells (from Sabo *et al.* [42]), splenocytes, the lymphoma cell line CH12, thymus, testis, and brain tissue (from ENCODE [43]). Significance is denoted by size (key upper left). **(e)** Average DNA methylation levels proximal to transcription factor motif binding sites. DNA methylation data are from 18 samples from 24 mice and 3 experiments, where 3 KO and 3 Ctl samples were analyzed for nB, GCB, and BMPC and two mice were pooled for GCB**.** Each cell type contained 4 female and 2 male (nB, GCB) or 4 male and 2 female (BMPC) mice split evenly between the genotypes.

Supplementary Figure 7

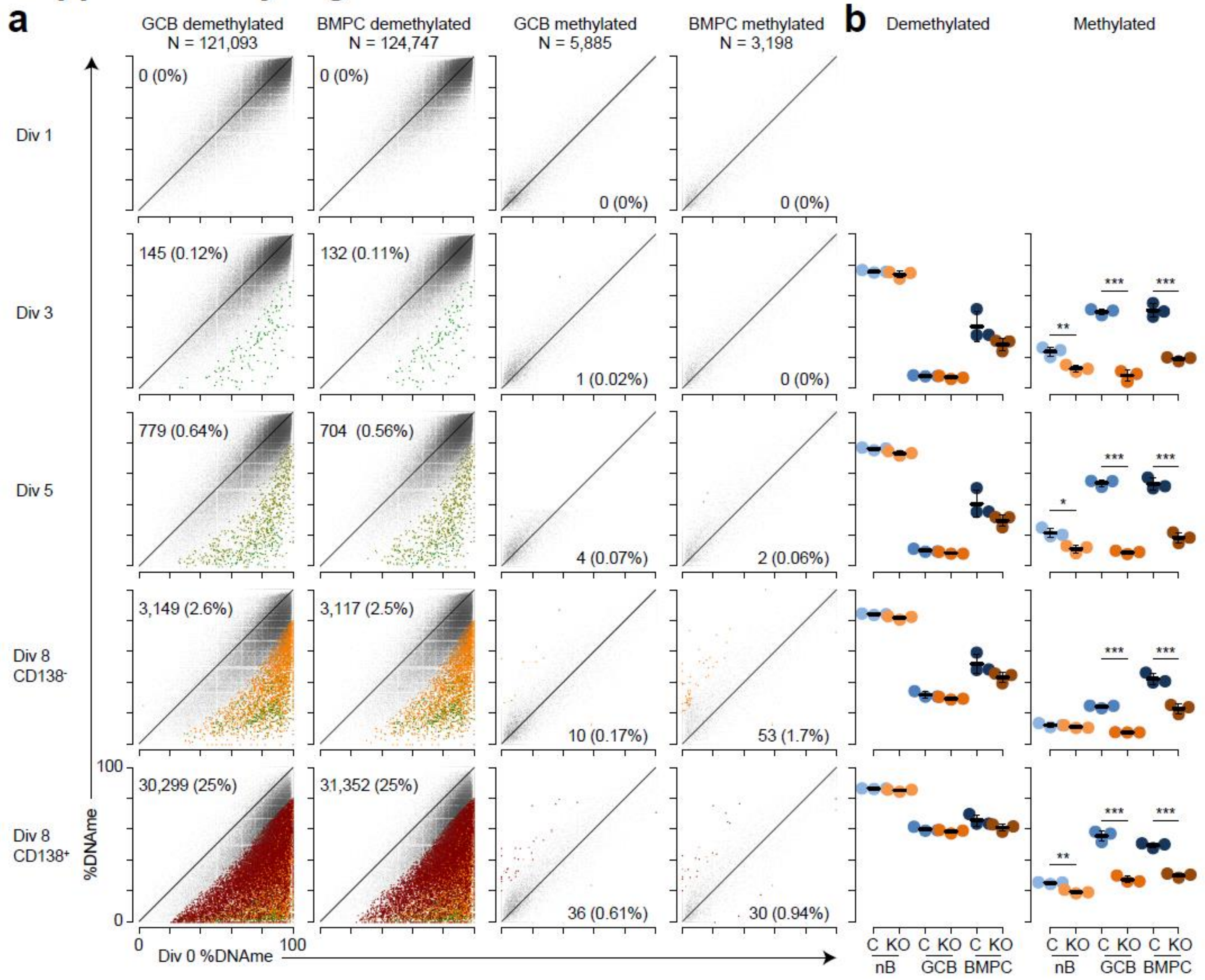

Supplementary Figure 7. Replication-coupled DNA hypomethylation. **(a)** Scatter plot of LPS-induced division-dependent DNA methylation changes for hypomethylated (left) and hypermethylated (right) loci found in Dnmt3-sufficient or –deficient B220$^+$PE$^+$GL7$^+$Fas$^+$ germinal center B cells (GCB) and CD138$^+$ bone marrow plasma cells (BMPC) as compared to B220$^+$GL7$^-$Fas$^-$ naive B cells (nB). The x-axis is the DNA methylation level in undivided (Div 0) B cells and the y-axis corresponds with divisions 1, 3, 5, 8 CD138$^-$, and 8 CD138$^+$ as previously described [6]. (**b**) Average methylation of division-specific demethylated and methylated loci are plotted in Dnmt3-sufficient and –deficient nB, GCB, and BMPC. **P* ≤0.05, ***P* ≤0.01, ****P* ≤0.001, Student's two-sided *t*-test with Tukey's post-hoc correction; mean and standard deviation are shown (**b**). Data are from 3 experiments and 24 mice where nB were isolated from 6 mice, GCB from 12 mice (2 mice were pooled per sample), and BMPC from 6 mice. Each cell type contained 4 female and 2 male (nB, GCB) or 4 male and 2 female (BMPC) mice split evenly between the genotypes.

Supplementary Figure 8

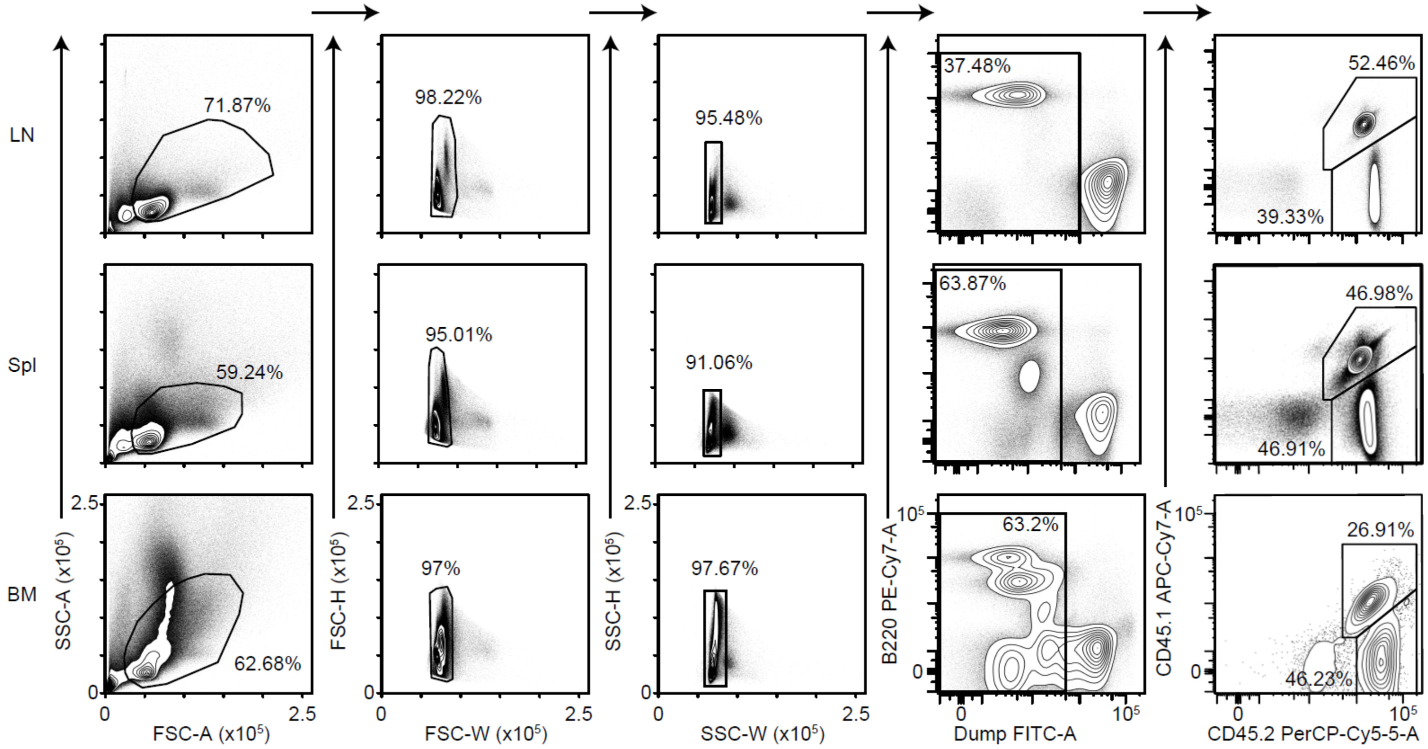

Supplementary Figure 8. Representative gating strategy. Plot of lymphocyte size gating for samples isolated from lymph nodes (LN; top), spleen (Spl; middle), and bone marrow (BM; bottom). Autofluorescent cells are removed by using a Dump channel (FITC) containing antibodies against CD11b, CD11c, CD90.2, and Ly6-G. Bone marrow chimera experiments utilized CD45.2 PerCP-Cy5.5 and CD45.1 APC-Cy7 to discriminate CD45.2$^+$ *Cd19*$^{cre/+}$*Dnmt3a*$^{fl/fl}$*Dnmt3b*$^{fl/fl}$ (KO) versus CD45.1$^+$ CD45.2$^+$ *Dnmt3a*$^{fl/wt}$*Dnmt3b*$^{fl/wt}$ (Control) cells. Figure 1 and Supplementary Figure 1 used a gating strategy without CD45.2 PerCP-Cy5.5 and CD45.1 APC-Cy7 whereas Figure 2 utilized CD45.2 PerCP-Cy5.5 and CD45.1 APC-Cy7.
