# Supplementary material for: B cell activation and plasma cell differentiation are inhibited by de novo DNA methylation
Source: Nat Commun. 2018 May 15;9:1900. doi: 10.1038/s41467-018-04234-4 (PMC5953949; doi:10.1038/s41467-018-04234-4)
Supplement: Supplementary file 2 — Description of Additional Supplementary Files [file 41467_2018_4234_MOESM2_ESM.pdf]

Description of Additional Supplementary Files

Supplementary Data 1. Differentially expressed genes between cell types and between Dnmt3-deficient and Dnmt3-sufficient cells of a given cell type.

Supplementary Data 2. Gene ontology analysis of differentially expressed genes in K-means clusters and genes differentially expressed between Dnmt3-deficient and Dnmt3-sufficient BMPC.

Supplementary Data 3. Differentially methylated loci between cell types and between Dnmt3-deficient and Dnmt3-sufficient cells of a given cell type.

Supplementary Data 4. Statistics for motifs enriched in differentially methylated loci and differentially accessible regions.

Supplementary Data 5. Differentially accessible peaks between cell types and between Dnmt3-deficient and Dnmt3-sufficient cells of a given cell type.

Supplementary Data 6. Differentially methylated loci proximal to differentially expressed genes.
